# Mortality, neurodevelopmental impairments, and economic outcomes after invasive group B streptococcal disease in early infancy in Denmark and the Netherlands: a national matched cohort study

**DOI:** 10.1016/S2352-4642(21)00022-5

**Published:** 2021-06

**Authors:** Erzsébet Horváth-Puhó, Merel N van Kassel, Bronner P Gonçalves, Brechje de Gier, Simon R Procter, Proma Paul, Arie van der Ende, Kirstine K Søgaard, Susan J M Hahné, Jaya Chandna, Stephanie J Schrag, Diederik van de Beek, Mark Jit, Henrik T Sørensen, Merijn W Bijlsma, Joy E Lawn

**Affiliations:** a Department of Clinical Epidemiology, Aarhus University, Aarhus N, Denmark; b Department of Neurology, Amsterdam Neuroscience, Amsterdam UMC, University of Amsterdam, Amsterdam, Netherlands; c Department of Paediatrics, Amsterdam UMC, University of Amsterdam, Amsterdam, Netherlands; d Netherlands Reference Laboratory for Bacterial Meningitis, Amsterdam UMC, University of Amsterdam, Amsterdam, Netherlands; e Maternal, Adolescent, Reproductive & Child Health Centre and Department of Infectious Disease Epidemiology, London School of Hygiene & Tropical Medicine, London, UK; f Centre for Infectious Disease Control, National Institute for Public Health and the Environment, Bilthoven, Netherlands; g The National Institute for Public Health and the Environment, University of Amsterdam, Amsterdam, Netherlands; h Department of Medical Microbiology and Infection Prevention, Amsterdam Infection and Immunity, Amsterdam, Netherlands; i Department of Clinical Microbiology, Aalborg University Hospital, Aalborg, Denmark; j Division of Bacterial Diseases, Centers for Disease Control and Prevention, Atlanta, GA, USA

## Abstract

**Background:**

Group B *Streptococcus* (GBS) disease is a leading cause of neonatal death, but its long-term effects have not been studied after early childhood. The aim of this study was to assess long-term mortality, neurodevelopmental impairments (NDIs), and economic outcomes after infant invasive GBS (iGBS) disease up to adolescence in Denmark and the Netherlands.

**Methods:**

For this cohort study, children with iGBS disease were identified in Denmark and the Netherlands using national medical and administrative databases and culture results that confirmed their diagnoses. Exposed children were defined as having a history of iGBS disease (sepsis, meningitis, or pneumonia) by the age of 89 days. For each exposed child, ten unexposed children were randomly selected and matched by sex, year and month of birth, and gestational age. Mortality data were analysed with the use of Cox proportional hazards models. NDI data up to adolescence were captured from discharge diagnoses in the National Patient Registry (Denmark) and special educational support records (the Netherlands). Health care use and household income were also compared between the exposed and unexposed cohorts.

**Findings:**

2258 children—1561 in Denmark (born from Jan 1, 1997 to Dec 31, 2017) and 697 in the Netherlands (born from Jan 1, 2000 to Dec 31, 2017)—were identified to have iGBS disease and followed up for a median of 14 years (IQR 7–18) in Denmark and 9 years (6–11) in the Netherlands. 366 children had meningitis, 1763 had sepsis, and 129 had pneumonia (in Denmark only). These children were matched with 22 462 children with no history of iGBS disease. iGBS meningitis was associated with an increased mortality at age 5 years (adjusted hazard ratio 4·08 [95% CI 1·78–9·35] for Denmark and 6·73 [3·76–12·06] for the Netherlands). Any iGBS disease was associated with an increased risk of NDI at 10 years of age, both in Denmark (risk ratio 1·77 [95% CI 1·44–2·18]) and the Netherlands (2·28 [1·64–3·17]). A history of iGBS disease was associated with more frequent outpatient clinic visits (incidence rate ratio 1·93 [95% CI 1·79–2·09], p<0·0001) and hospital admissions (1·33 [1·27–1·38], p<0·0001) in children 5 years or younger. No differences in household income were observed between the exposed and unexposed cohorts.

**Interpretation:**

iGBS disease, especially meningitis, was associated with increased mortality and a higher risk of NDIs in later childhood. This previously unquantified burden underlines the case for a maternal GBS vaccine, and the need to track and provide care for affected survivors of iGBS disease.

**Funding:**

The Bill & Melinda Gates Foundation.

**Translations:**

For the Dutch and Danish translations of the abstract see Supplementary Materials section.

## Introduction

Group B *Streptococcus* (GBS), specifically *Streptococcus agalactiae,* is a leading cause of invasive bacterial disease in neonates and young infants (0–89 days).^1^, ^2^ Estimates suggest that the burden affects all regions worldwide, with more than 21·7 million pregnant women affected, 55 000 (uncertainty range [UR] 12 000–104 000) stillbirths, 319 000 (145 000–653 000) infant cases of invasive GBS (iGBS) disease, and 90 000 (36 000–169 000) deaths in infants younger than 3 months annually.^3^

Assessing the total burden associated with iGBS disease is crucial to design strategies for service provision and prevention, including evaluation of the potential benefit of maternal GBS vaccines that are currently in development.^4^, ^5^, ^6^ However, there are important data gaps, notably regarding the long-term health and economic outcomes of individuals after recovering from iGBS disease. Patients who survive iGBS disease might be at risk of increased mortality later in life and of long-term neurodevelopmental impairments (NDIs), including intellectual, motor, vision, and hearing impairments. A meta-analysis of NDI after iGBS disease included 18 small studies (a total of 453 participants) only after meningitis.^7^ This result highlights a key data gap regarding the long-term adverse outcomes after sepsis, which represents the majority of patients with iGBS infection.^7^ Data for patients older than 2 years were few and collected primarily in the 1970s. Most studies did not include a comparator cohort and, importantly, did not consider gestational age, a major driver of NDI, in analyses.^7^, ^8^, ^9^, ^10^, ^11^, ^12^, ^13^, ^14^ Hence, robust, up-to-date data on impairments that manifest in childhood or adolescence, and especially after GBS sepsis, are inadequate. Economic outcome data are even more scarce, with only one small study of patients up to the age of 2 years from the UK.^15^

**Research in context**

**Evidence before this study**

Invasive group B Streptococcus (iGBS) infection is a leading cause of neonatal mortality and morbidity worldwide, but important knowledge gaps exist regarding the long-term outcomes of patients who survive iGBS disease. A 2017 systematic review and meta-analysis of outcomes after iGBS found only 18 small studies (n=453 patients) assessing neurodevelopmental impairment (NDI), almost exclusively before the age of 2 years and without comparator groups. All studies focused on NDIs after meningitis, and not after sepsis, which is more common. Only one study described the economic costs linked to iGBS disease following children up to the age of 2 years. In November, 2020, Pubmed was searched with terms similar to the 2017 review relating to “Streptococcus agalactiae [MesH]”, “group B streptococcus”, and “disability” “impairment” with no restrictions for date and language. We did not identify any new published studies meeting the inclusion criteria.

**Added value of this study**

To our knowledge, our study is the first to quantify the major enduring effect of iGBS disease on health outcomes of survivors up until adolescence, including mortality, NDI, health-care use, and household income. We used linked national health-care databases from Denmark and the Netherlands to generate cohorts with more than 24 000 children included. In Denmark, the risk of a diagnosis of moderate or severe NDI by the age of 10 years was two times higher among children with history of iGBS disease compared with unexposed children. Similarly, children in the Netherlands with a history of iGBS disease were twice as likely to require educational support, including enrolment in special needs schools, compared with unexposed children. Both a history of GBS sepsis and GBS meningitis were associated with NDIs and a need for educational support. Throughout childhood, frequencies of outpatient clinic visits and hospital admissions were higher in children who had a history of iGBS disease than in those without iGBS disease during early infancy. Although GBS meningitis was associated with higher mortality in children younger than 5 years in both Denmark and the Netherlands, increased mortality during or after GBS sepsis was observed only in the Netherlands.

**Implications of all the available evidence**

Our findings show that the burden of iGBS disease is unacceptably high, despite intrapartum prophylactic antibiotic strategies and advanced neonatal care in high-income countries. The burden of iGBS disease is higher in lower-income and middle-income countries, where most at-risk births and the majority of cases occur. Improved preventive interventions, such as maternal GBS vaccines, are urgently needed.

In this study, we constructed cohorts of children who had had iGBS infection in early infancy using national databases in Denmark and the Netherlands. Our objectives were to: (1) describe children with iGBS infection; (2) examine excess mortality during and after iGBS disease in comparison with the unexposed cohorts; (3) estimate the risk of NDI up until adolescence, comparing exposed cohorts with unexposed cohorts; and (4) assess the effects of iGBS disease on long-term health-care use and household income.

## Methods

### Study design and participants

We did a nationwide matched cohort study using Danish population-based medical and administrative registries (born Jan 1, 1997–Dec 31, 2017) and Dutch population registries (born Jan 1, 2000–Dec 31, 2017), up to 21 years of age in Denmark and up to 18 years of age in the Netherlands. Exposed children were defined as having a history of iGBS disease (sepsis, meningitis, or pneumonia) by the age of 89 days. Unexposed children without iGBS disease were randomly selected and matched at a ratio of 10:1 on sex, birth month and year, and gestational age (<28 weeks, 28–36 weeks, and ≥37 weeks) to each exposed child.

In Denmark, the Medical Birth Registry and the Danish National Patient Registry were used to identify children with iGBS disease. The Danish National Patient Registry contains records of all admissions to Danish non-psychiatric hospitals since 1977, and outpatient clinic and emergency room visits since 1995. GBS meningitis, sepsis, and pneumonia were defined on the basis of discharge diagnoses with the use of International Classification of Diseases (ICD)-10 codes (appendix 3 p 10).^16^, ^17^, ^18^ To assess the comprehensiveness of discharge ICD-10 codes in identifying iGBS disease in Denmark, we compared the GBS cohort from 1997–2003 with the North Denmark Bacteraemia Research Database, which has registered all patients from the north Denmark region diagnosed with culture-proven bacteraemia.^19^ The unexposed cohort was sampled from the Danish Medical Birth Registry and the Danish Civil Registration System.^17^ The index date corresponded with the birth date.

In the Netherlands, patients with iGBS disease were identified from the Netherlands Reference Laboratory for Bacterial Meningitis, which receives approximately 90% of isolates cultured from the blood or cerebrospinal fluid (CSF) of infants with invasive infection from microbiology laboratories.^20^, ^21^ All infants with CSF or blood culture positive for GBS, or both, were eligible for inclusion. Meningitis was defined as positive CSF culture or both positive CSF and blood cultures. Sepsis was defined as a positive blood culture only. The unexposed cohort was randomly selected with the use of the PeriNed perinatal registry (a database covering approximately 99% of all births in the Netherlands) and the Municipal Personal Records Database (which records mortality data).^22^ More information on the databases in both countries is provided in appendix 3 (pp 5–9). The study was approved by the Danish Data Protection Agency (record number 2015-57-0002). In the Netherlands, the study protocol (EPI-408) was submitted to the Centre for Clinical Expertise at the National Institute for Public Health and the Environment. The study protocol was exempted from further approval by an ethical research committee, according to Dutch law for medical research involving human subjects.

### Procedure

The first objective was to examine the clinical characteristics of children with iGBS disease. For each child, information on sex, year and month of birth, multiplicity, gestational age, and birthweight was obtained from population registries (appendix 3 pp 10, 11). In Denmark, the age of iGBS onset was calculated from the first admission to hospital date for iGBS disease. In the Netherlands, the first reported date of illness, mostly the first date a culture was taken (in 97·4% of patients), was used to calculate the age at onset. iGBS disease was categorised as either early onset (0–6 days old) or late onset (7–89 days old).

The second objective was to establish mortality during and after iGBS disease. All-cause mortality during the first 3 months and 5 years of life were assessed, on the basis of data from the Danish Civil Registration System^17^ and the Dutch Municipal Personal Records Database. In Denmark, mortality during hospital admission for iGBS disease was also examined (data were not available in the Netherlands).

The third objective was to look at the risk of NDIs. NDIs were defined differently in the two cohorts. In Denmark, we obtained information on NDIs from the Danish National Patient Registry^18^ using ICD-10 codes for mental, behavioural, and nervous system disorders. We assessed the motor, hearing, vision, cognitive, and social or behavioural domains, and categorised impairments by severity (mild, moderate, or severe; definitions specified in appendix 3 pp 11–13). Overall, an NDI was defined as an impairment in any domain, and a multidomain NDI as an impairment in more than one domain.^9^

In the Netherlands, special educational support was used as a surrogate marker for an NDI. National databases covering primary school registration and special education were used to identify children who received education in special needs schools (considered as a moderate or severe NDI) or who received additional support in regular schools (considered as a mild NDI).

The fourth objective was to examine long-term health-care use and household income. In Denmark, data on the number of days of hospital care, hospital admissions, and outpatient clinic visits were obtained from the Danish National Patient Registry and calculated for each year of follow-up (data were not available in the Netherlands). The outpatient clinic visits included contact with hospital-based (ambulatory) specialty clinics but not with private practice specialists or general practitioners.

In Denmark, data on individual income from the Income Statistics Register^23^ were used to derive annual household income by summing the gross income of each cohort member's parents. This number was then converted to Euros.^24^ In the Netherlands, household income was ascertained from a registry database of standardised disposable household income after adjustment for family size and taxes. Data on the percentage of gross household income from welfare payments were also obtained from the same registry database. In both countries, incomes were adjusted to the 2018 currency values with the use of the country-specific World Bank gross domestic product deflator.^25^ Of note, household income was assessed for each year after the first year in which the invasive iGBS episode occurs, not at birth, and was not adjusted for baseline income. Rather, the household income of the exposed group was compared with the household income of the families with unexposed children who were matched to the exposed group.

### Statistical analysis

All children were followed up from birth until death, emigration, or until the end of the study (Dec 31, 2017), whichever came first. We computed the mortality risk during the acute iGBS episode, first 3 months, and first 5 years of life (objective 2). Survival curves were plotted with the use of the Kaplan-Meier technique (appendix 3 pp 20, 21). Mortality rates were computed per 1000 person-years of follow-up. Hazard ratios (HRs), adjusted for the matching factors, were computed with the use of Cox proportional hazards regression and compared children with iGBS disease with unexposed children. The proportional hazards assumption was assessed graphically with the use of log–log plots and was found to be appropriate.

For NDI analyses (objective 3), cohorts were restricted to children who survived the first 3 months of life. Risks of overall, domain-specific, and multidomain NDI, as well as special educational needs, were assessed at different ages (Denmark, 5, 7, 10, 15, and 20 years; the Netherlands, 5, 7, 10, and 11 years). We included only children followed up until at least the corresponding cutoff age. To quantify the association between iGBS disease and NDI, we used a modified Poisson regression with robust variance estimators to estimate risk ratios (RRs) adjusted for the matching variables.^26^

Health-care use in Denmark was assessed annually (objective 4), and compared between cohorts with the use of negative binomial regression models. The average household income was also described. Sensitivity analyses compared exposed cohorts with a second unexposed cohort matched on sex and birth month and year, but not on gestational age categories, to evaluate the relative effect of iGBS and potential contribution of prematurity. In Denmark, data on gestational age were missing for 28 neonates, therefore they were excluded from the primary analyses. In Denmark, statistical analyses were done using SAS version 9.4, and in the Netherlands, they were done using SPSS Statistics version 25.0 and STATA version 16.

### Role of the funding source

The funder of the study had no role in in study design, data collection, data analysis, data interpretation, or writing of the report.

## Results

**Figure 1:**
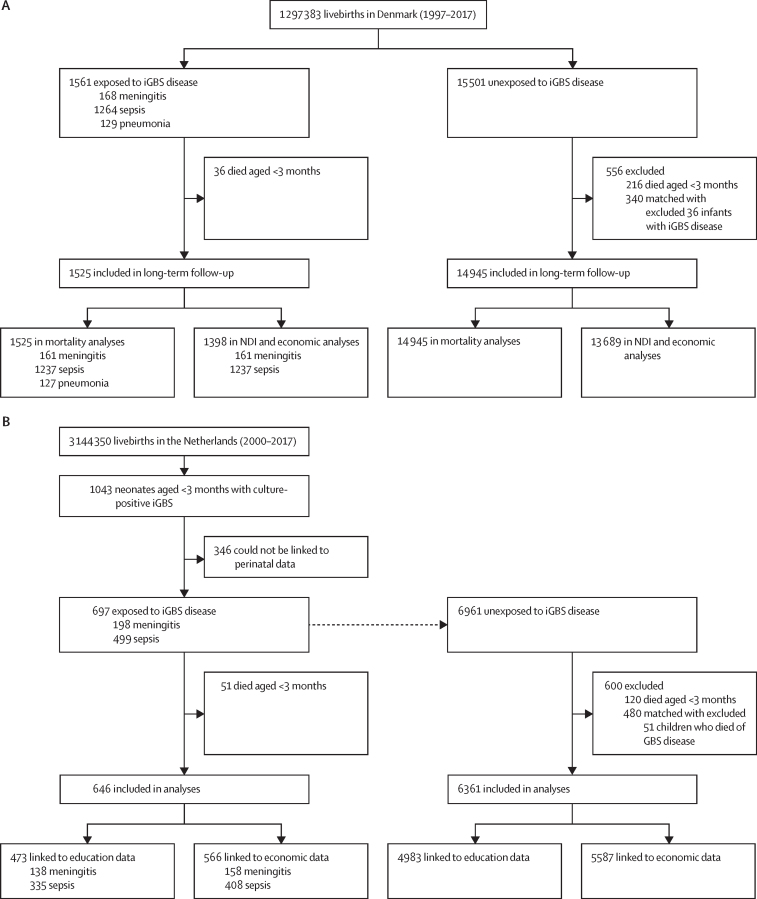
Study flowcharts showing children with iGBS disease and gestational age-matched unexposed cohorts in Denmark (A) and the Netherlands (B) Of 1525 iGBS cases, 1398 were included in the NDI and economic analyses (after including only those who survived iGBS and excluding pneumonia). This cohort was further restricted in the NDI analyses to those who were alive at age of 5 years (n=1293), 7 years (n=1179), and 10 years (n=969). iGBS=invasive group B *Streptococcus*. NDI=neurodevelopmental impairments.

Source populations encompassed 1 297 383 livebirths (Jan 1, 1997–Dec 31, 2017) in Denmark and 3 144 350 livebirths (Jan 1, 2000–Dec 31, 2017) in the Netherlands. Overall, we identified 2258 children with a history of iGBS disease, to whom 22 462 children with no history of iGBS disease were matched (figure 1). Children with iGBS disease were followed up for a median of 14 years (IQR 7–18) in Denmark and 9 years (6–11) in the Netherlands. In Denmark, 1295 (83%) of 1561 exposed children had iGBS with onset in the first 72 h after birth, compared with 392 (56%) of 697 in the Netherlands. Most exposed children had a history of GBS sepsis (table 1), occurring most often in the first 72 h of life (1122/1264 [88·8%] in Denmark and 335/499 [67·1%] in the Netherlands). A lower percentage of children had GBS meningitis (table 1), often after the first week of life (88/168 [52·4%] in Denmark and 113/198 [57·1%] in the Netherlands). The early onset versus the late onset ratio of cases differed between countries and was stable over time (table 2).

**Table 1:** Characteristics of children with iGBS disease and members of a matched comparison cohort in Denmark and the Netherlands

		**Denmark (1997–2017)**		**Netherlands (2000–2017)**	
		Exposed cohort (n=1561)	Unexposed cohort (n=15 501)	Exposed cohort (n=697)	Unexposed cohort (n=6961)
iGBS clinical syndrome					
	Meningitis	168 (10·8%)	NA	198 (28·4%)	NA
	Sepsis	1264 (81·0%)	NA	499 (71·6%)	NA
	Pneumonia	129 (8·3%)	NA	..	NA
Sex					
	Female	700 (44·8%)	6942 (44·8%)	309 (44·3%)	3081 (44·3%)
	Male	861 (55·2%)	8559 (55·2%)	388 (55·7%)	3880 (55·7%)
Gestational age					
	<28 weeks	53 (3·4%)	421 (2·7%)	30 (4·3%)	291 (4·2%)
	28–36 weeks	274 (17·6%)	2740 (17·7%)	151 (21·7%)	1510 (21·7%)
	≥37 weeks	1234 (79·1%)	12 340 (79·6%)	516 (74·0%)	5160 (74·1%)
Multiplicity					
	Singleton	1460 (93·5%)	14 242 (91·9%)	649 (93·1%)	6385 (91·7%)
	Twins	93 (6·0%)	1213 (7·8%)	44 (6·3%)	554 (8·0%)
	Higher order	8 (0·5%)	46 (0·3%)	<5 (<0·7%)	21 (0·3%)
Birthweight (kg)		3·5 (2·9–3·9)	3·4 (2·9–3·8)	3·3 (2·7–3·7)	3·3 (2·7–3·7)
Maternal age (years)		30·4 (27·0–33·7)	30·4 (27·2–33·8)	30·4 (26·7–33·8)	31·1 (27·8–34·4)

Numbers are presented as n (%) or median (IQR). In the Netherlands, the number of recruited children with a history of iGBS disease only partly reflects the country-level incidence of iGBS disease, because cases were restricted to those that could be linked between several datasets. iGBS=invasive group B *Streptococcus*. NA=not applicable.

**Table 2:** Prevalence of early-onset and late-onset GBS disease across different time frames in the study period

	**Denmark**		**Netherlands**	
	Early onset (n=1327)	Late onset (n=234)	Early onset (n=445)	Late onset (n=252)
1997–99	312 (89·4%)	37 (10·6%)	..	..
2000–05	437 (86·4%)	69 (13·6%)	109 (66·9%)	54 (33·1%)
2006–11	310 (80·9%)	73 (19·1%)	138 (65·7%)	72 (34·3%)
2012–17	268 (83·0%)	55 (17·0%)	198 (61·1%)	126 (38·9%)

Numbers are presented as n (%). Early-onset disease was defined as age 0–6 days and late-onset disease as age 7–89 days. GBS=invasive group B *Streptococcus*.

Mortality risk in the exposed cohort in the first 89 days of life was 2·3% (95% CI 1·7–3·2%) in Denmark and 7·6% (5·6–9·5%) in the Netherlands. In the unexposed cohort, mortality was considerably lower: 1·7% (1·5–1·9%) in Denmark and 1·7% (1·4–2·1%) in the Netherlands. To estimate the potential contribution of prematurity to these mortality risks, we calculated mortality in the unexposed cohorts that were not matched for gestational age (0·3% [95% CI 0·2–0·4%] in Denmark and 0·4% [0·3–0·6%] in the Netherlands), which was lower than those matched for gestational age. Preterm infants (gestational age <37 weeks) with a history of iGBS disease had a higher mortality rate during the first 3 months of life than did infants carried to term (gestational age ≥37 weeks). In Denmark, 6·7% (95% CI 4·5–10·0%) of preterm and 1·1% (0·7–1·9%) of term children with iGBS disease died during the study period. In the Netherlands, 15·5% (10·0–20·4%) of preterm children and 5·0% (3·3–7·1%) of term children died. In Denmark, 33 of 1561 children with iGBS (2·1%) died during their hospital stay for iGBS.

In the Netherlands, the 5-year mortality rate was higher in the exposed cohort than the unexposed cohort (table 3). 5-year HRs in Denmark were 4·08 (95% CI, 1·78–9·35) for iGBS meningitis, 0·79 (0·54–1·16) for iGBS sepsis, and 0·59 (0·14–2·55) for iGBS pneumonia. In the Netherlands, the corresponding HRs were 6·73 (3·76–12·06) for iGBS meningitis and 3·23 (2·19–4·76) for iGBS sepsis (table 3).

**Table 3:** Mortality rates and HRs of children with iGBS disease and unexposed cohort members, by age and clinical syndrome

	**Any iGBS disease**				**iGBS meningitis**				**iGBS sepsis**			
	Exposed mortality rate (95% CI)	Unexposed mortality rate (95% CI)	Mortality rate difference (95% CI)	HR (95% CI)	Exposed mortality rate (95% CI)	Unexposed mortality rate (95% CI)	Mortality rate difference (95% CI)	HR (95% CI)	Exposed mortality rate (95% CI)	Unexposed mortality rate (95% CI)	Mortality rate difference (95% CI)	HR (95% CI)
**Denmark**												
0–89 days	96·6 (65·0 to 128·1)	68·9 (60·4 to 77·3)	27·7 (−4·9 to 60·4)	0·97 (0·68 to 1·37)	177·1 (45·9 to 308·3)	34·6 (16·5 to 52·7)	142·5 (10·1 to 274·9)	3·45 (1·37 to 8·66)	89·3 (55·6 to 123·0)	72·6 (62·9 to 82·2)	16·8 (−18·3 to 51·8)	0·83 (0·56 to 1·24)
0–5 years	5·6 (3·9 to 7·4)	4·1 (3·6 to 4·5)	1·5 (−0·3 to 3·3)	0·96 (0·69 to 1·34)	12·6 (4·4 to 20·8)	2·1 (1·1 to 3·2)	10·5 (2·2 to 18·8)	4·08 (1·78 to 9·35)	5·0 (3·2 to 6·8)	4·3 (3·8 to 4·9)	0·7 (−1·2 to 2·6)	0·79 (0·54 to 1·16)
**Netherlands**												
0–89 days	319·4 (231·8 to 407·1)	71·9 (59·0 to 84·8)	247·6 (158·9 to 336·2)	4·05 (2·92 to 5·62)	424·5 (233·6 to 615·4)	48·3 (28·5 to 68·0)	376·3 (184·4 to 568·2)	7·82 (4·25 to 14·38)	278·5 (182·0 to 375·0)	81·3 (65·2 to 97·5)	197·2 (99·3 to 295·0)	3·17 (2·12 to 4·72)
0–5 years	19·4 (14·2 to 24·7)	4·6 (3·8 to 5·4)	14·9 (9·6 to 20·2)	3·97 (2·89 to 5·46)	24·1 (13·4 to 34·9)	3·3 (2·1 to 4·5)	20·8 (9·9 to 31·7)	6·73 (3·76 to 12·06)	17·6 (11·7 to 23·5)	5·1 (4·1 to 6·1)	12·4 (6·4 to 18·4)	3·23 (2·19 to 4·76)

Mortality rates are expressed in events per 1000 child-years; hazard ratios are adjusted for matching variables (ie, sex, year of birth, and gestational age). HR=hazard ratio. iGBS=invasive group B *Streptococcus*.

**Figure 2:**
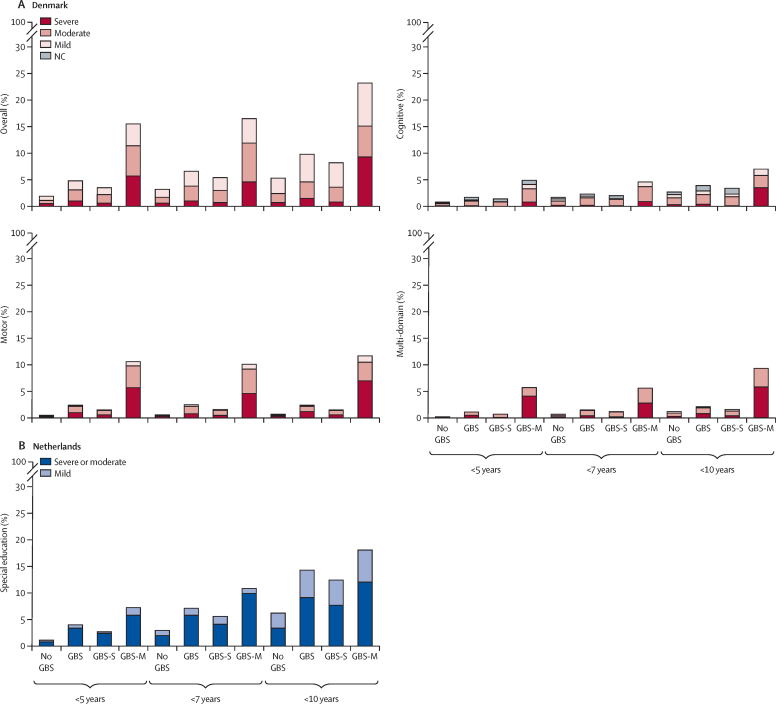
Proportion of children with NDIs among those with invasive GBS disease compared with unexposed children matched on gestational age in Denmark (A) and the Netherlands (B) (A) Proportions of children with NDIs of different severities at different ages in the Danish study population are shown for the exposed and unexposed cohorts. For these four panels, the NDI outcomes include: cognitive domain, motor domain, overall NDI, and multi-domain NDIs. Of the 1525 children who survived GBS disease in Denmark, 1293 (by age 5 years) and 969 (by age 10 years) were included in analyses of long-term impairment. (B) The proportion of children with NDIs in the study population from the Netherlands. Children enrolled in special needs schools are presented as having moderate or severe NDI. 489 of the 646 patients who survived GBS in the Netherlands reached the age of mandatory education; 16 (3·2%) were not found in the national database and were considered lost to follow-up. The diagnosis of NDIs was defined cumulatively—namely, that each child was considered to have impairments based on all follow-up information available up to the relevant age limit. GBS=group B *Streptococcus*. GBS-M=GBS meningitis. GBS-S=GBS sepsis. NC=not categorised. NDIs=neurodevelopmental impairments.

Of the 1525 children who survived iGBS disease in Denmark, 1293 were included in analyses of long-term impairment by the age of 5 years and 969 by the age of 10 years. In the Netherlands, 489 of the 646 patients who survived iGBS reached the age of mandatory education (5 years); 16 (3·2%) of them were not found in the national database and were considered lost to follow-up (figure 1B). iGBS was associated with an overall risk of NDI by age 10 years (RR 1·77 [95% CI 1·44–2·18] in Denmark and 2·28 [1·64–3·17] in the Netherlands; appendix 3 pp 15, 16). In Denmark, 45 (4·6%) of 969 of patients who survived iGBS disease had moderate or severe NDI at 10 years, compared with 234 (2·5%) of 9519 in the unexposed cohort (RR 1·82 [95% CI 1·33–2·49]). This proportion was even lower in the unexposed cohort not matched by gestational age than the matched unexposed cohort (2·1%). A similar pattern was observed in the Netherlands: among patients who survived iGBS, 27 (7·1%) of 380 received some form of special educational support by the age of 7 years and 36 (14·3%) of 252 by 10 years, compared with 111 (2·9%; aged 7 years) of 3776 and 157 (6·2%; aged 10 years) of 2527 in the unexposed cohort, and 92 (1·9%; aged 7 years) of 4844 and 185 (5·3%; aged 10 years) of 3495 in the unexposed cohort not matched by gestational age. Children with a history of GBS meningitis had a higher frequency of impairment than did those previously diagnosed with GBS sepsis only (figure 2). Both children with a history of GBS meningitis and GBS sepsis had a higher NDI risk than did the unexposed group (figure 2; appendix 3 pp 15, 16). Similarly, both early-onset and late-onset iGBS were associated with an increased NDI risk (appendix 3 pp 15, 16).

Consistently higher frequencies of moderate and severe motor and cognitive impairments were observed in children who survived iGBS disease than in unexposed children (appendix 3 pp 15, 16). In Denmark, the co-occurrence of moderate or severe impairment in two or more domains by the age of 10 years was observed in 19 (2·0%) of 969 patients who survived iGBS disease, and in 90 (0·9%) of 9519 unexposed children (RR 1·98 [95% CI 1·22–3·21]; appendix 3 p 15).

**Figure 3:**
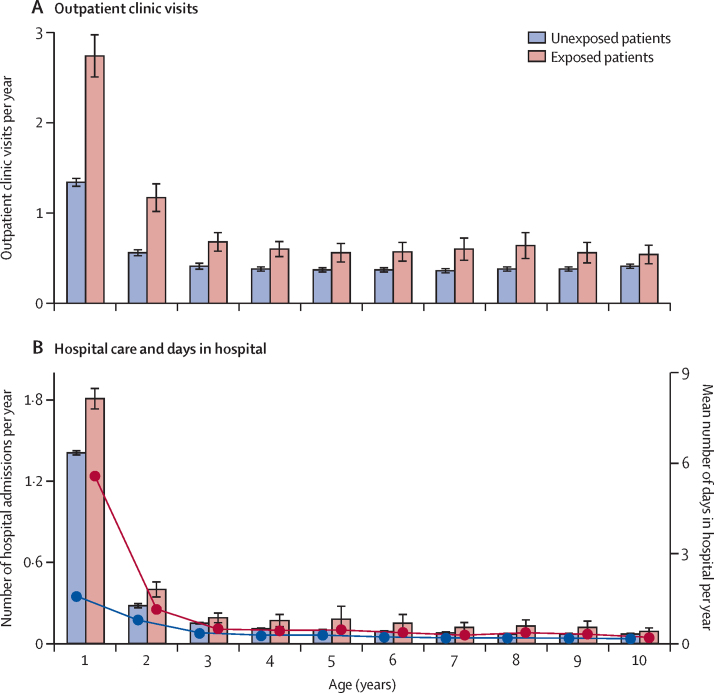
Health-care use among children with invasive GBS disease compared with unexposed children that were matched by gestational age in Denmark (A) Outpatients clinic visits. (B) Number of hospital admissions (bars) and mean number of days in hospital (red line corresponds to exposed children; blue line corresponds to unexposed children). 95% CIs of age-specific means are presented for each bar. Hospital admissions for acute GBS were excluded in the analysis of number of days of hospitalisation in the first year of life. GBS=group B Streptococcus disease.

In Denmark, patients who survived iGBS disease had higher rates of outpatient clinic visits, hospital admissions, and a higher number of days in hospital per year than did unexposed children (figure 3). In negative binomial regression models accounting for differences in follow-up time, a history of iGBS disease was associated with more frequent outpatient clinic visits (incidence rate ratio 1·93 [95% CI 1·79–2·09], p<0·0001) and hospital admissions (1·33 [1·27–1·38], p<0·0001) in children 5 years or younger. Similar results were obtained in analyses of children aged 10 years or younger for outpatient clinic visits (1·83 [1·67–2·00], p<0·0001) and hospital admissions (1·43 [1·37–1·50], p<0·0001). No differences in household income were observed (appendix 3 p 22). Dutch data also showed no clear difference in the receipt of welfare payments (appendix 3 p 23).

## Discussion

We observed that children with a history of iGBS disease, particularly those with meningitis, had a higher mortality rate than did children without a history of iGBS disease. In Denmark, NDIs, including moderate and severe disabilities, were more frequent in children who had survived iGBS disease. In the Netherlands, these children required more educational support. The rates of use of health care were also higher in children who had iGBS disease. However, the average household income did not differ between the families of exposed and unexposed children. Overall, our results show that iGBS disease continues to affect children and their families well beyond the neonatal period.

Both in Denmark and the Netherlands, mortality was higher during early infancy for children with iGBS disease than it was for unexposed children. However, mortality in the first months of life in Denmark was considerably lower than in the Netherlands and lower than the mortality reported for other high-income countries; for example, in one study in the UK, the case fatality rate was 6·2%,^27^ and in a systematic review, it was 4·7% (95% CI 3·3–6·1%) during an iGBS infection.^28^ This lower mortality rate could represent a real difference in mortality or more likely could be related to the differences in the study populations. In terms of missed cases, it is possible that in Denmark, ICD-based case identification selectively missed some GBS infections on the day of birth and these cases might have a higher mortality rate. In addition, the Danish cohort included patients with GBS disease who only had pneumonia, which might be associated with lower mortality.^29^ These patients were not included in the Netherlands' system, which diagnoses patients on the basis of positive cultures. Because of the small number of children with a diagnosis of GBS pneumonia in Denmark, we might have been underpowered to compare the mortality risk during or after GBS pneumonia. It is important to note that the Danish cohort potentially included some children with GBS infections not confirmed by culture. We validated the discharge diagnosis codes for a subgroup of children diagnosed by culture in the north region of Denmark (1997–2013). In the Netherlands, only culture-proven infections were included. This selectiveness might have contributed to the observed difference, because a higher mortality rate has been reported in culture-proven (∼7%) versus clinically probable (∼1%) sepsis cases,^30^ although the definition of ICD-coded and clinically probable cases could differ.

Maternal GBS colonisation is a known risk factor for both prematurity and iGBS infection^31^ and there might be an increased risk of iGBS disease in preterm infants.^32^ Adjustment for gestational age reduced the association between mortality and iGBS, and the adjusted HRs for children with a history of GBS meningitis were lower (4·08 in Denmark and 6·73 in the Netherlands) compared with those in unadjusted analyses (22·88 and 14·77; appendix 3 p 14).

Because few deaths were observed after the first 3 months of life, we were unable to analyse mortality among children who survived the acute episode. Furthermore, because of the low numbers, we were unable to assess differences in the mortality rates of children with a history of iGBS pneumonia. The uncertainty in this clinical diagnosis because of the frequent co-occurrence of sepsis emphasises the importance of doing prospective studies on patients with iGBS pneumonia with clear laboratory and clinical criteria.

Data on NDI after iGBS disease are sparse. We found that patients who survived iGBS sepsis or meningitis are at a considerably higher risk of NDI than are unexposed children, regardless of the outcome or method used to identify disability. Our longitudinal data also showed a progressive increase in NDI frequency as children grow up, which illustrates the importance of long-term follow-up to capture the burden of iGBS disease. In a systematic review of 18 studies on NDIs after iGBS disease, a 18% risk of moderate or severe NDI was reported in patients who survived GBS meningitis (n=453) followed up for a median of 18 months or longer. The few studies identified that had follow-up data in children older than 2 years were primarily done in the 1970s,^33^, ^34^ and were limited by small sample sizes. Data on NDI in patients who survived GBS sepsis were few. Our study addressed these major data gaps by assessing NDI up until adolescence, and by including data from more than 1500 children who survived iGBS sepsis. The results for iGBS sepsis have major implications for estimating the global burden of iGBS. An excess risk of disability after GBS sepsis, although lower than that after meningitis, might be a higher attributable risk given that GBS sepsis is much more common. Additionally, since this is a lifetime risk of disability, years lived with disability and disability-adjusted life-years could be considerable. Hence our findings will be important when considering the potential effect and cost-effectiveness of interventions, such as intrapartum antibiotic prophylaxis and maternal vaccination.

Families' economic circumstances might plausibly be affected by the severe illness of a child and subsequent NDIs might result in costs both to health-care systems and to families. The only previous study assessing the economic costs of iGBS disease (n=138) reported two-times higher health and social care costs over the first 2 years of life.^15^ Our results, based on an at least five-times longer follow-up than the previous study, are consistent. In Denmark, patients who survived iGBS had a higher rate of health-care use. Furthermore, we found that a higher frequency of health-care visits persisted until at least the age of 10 years. However, we did not find a measurable effect on household income in either country. Nevertheless, similar average incomes in exposed and unexposed groups do not preclude an effect on families' expenditure to support their child. It is probable that the national health systems in both countries covered or compensated most of the direct health-care-related costs. But, given the much higher health-care use and the need for special educational support, iGBS disease could substantially affect family expenditure and income in countries with less access to health care and social support.

Our study has several strengths, notably a large sample size, a large number of unexposed comparison children, a multinational design, long-term follow-up up until adolescence, and high-quality data. However, the study also has limitations. In appendix 3 p 18, we list the differences in the capture of iGBS disease and outcome data between the two countries, and potential biases. Although differences in case selection (diagnosis-based *vs* culture-based) might explain some of the disparities in mortality outcomes, the comparison with a cohort that is unmatched by gestational age suggests that exposure misclassification is not the only explanation. NDI data in Denmark were restricted to patients diagnosed in a hospital setting, leading to a probable underestimation of vision and hearing problems. Another limitation was our use of special education support as a proxy for NDI in the Netherlands. Our estimates of special education needs after GBS meningitis are consistent with the results of the meta-analysis on NDIs after GBS disease by Kohli-Lynch and colleagues^7^ and we estimated relative risk using a matched unexposed cohort; however, we cannot rule out that children with NDIs might have been missed in both exposed and unexposed groups. Indeed, if the severity of the NDI was too high to permit school attendance, these children could have been missed, leading to the underestimation of NDIs.

iGBS disease, particularly meningitis, during early infancy has a persistent effect on the lives of affected children and their families. In low-income and middle-income countries, the long-term effect might be even greater, and future studies should address outcomes in such countries. Furthermore, in Denmark and the Netherlands, risk-based policies on intrapartum antibiotic prophylaxis were adopted nearly two decades ago. Despite this, the high risk of fatal outcomes and NDIs suggest that additional preventive measures, such as a maternal GBS vaccine to protect infants, are needed to minimise associated morbidity and mortality long after an infection with iGBS.

## Data sharing

Although local data governing bodies in Denmark and in the Netherlands do not authorise individual level data from these electronic cohorts to be shared, datasets with selected aggregated data are available from the corresponding author on reasonable request.

## Declaration of interests

AvdE received grants from Pfizer for research on pneumococcal infections (investigator initiated project IIR WI173197) and for research on meningococcal infections (investigator initiated project IIR WI242174), outside the submitted work; participated in the Advisory Boards of Pfizer, GlaxoSmithKline, and Sanofi-Pasteur; and did consultancy activities for GlaxoSmithKline and Merck Sharp & Dohme (fees paid to Amsterdam University Medical Center). HTS reports that the Department of Clinical Epidemiology is involved in studies with institutional funding from regulators and from various pharmaceutical companies, as research grants to and administered by Aarhus University. None of these studies are related to the current study. All other authors declare no competing interests.

## Supplementary Material

Dutch translation of the abstract

Danish translation of the abstract
