# Supplementary material for: Mortality, neurodevelopmental impairments, and economic outcomes after invasive group B streptococcal disease in early infancy in Denmark and the Netherlands: a national matched cohort study
Source: Lancet Child Adolesc Health. 2021 Jun;5(6):398–407. doi: 10.1016/S2352-4642(21)00022-5 (PMC8131199; doi:10.1016/S2352-4642(21)00022-5)
Supplement: Dutch translation of the abstract [file mmc1.pdf]

THE LANCET
Child & Adolescent Health

## Supplementary appendix 1

This translation in Dutch was submitted by the authors and we reproduce it as supplied. It has not been peer reviewed. *The Lancet*'s editorial processes have only been applied to the original in English, which should serve as reference for this manuscript.

Deze vertaling in het Nederlands werd door de auteurs toegevoegd en we reproduceren de tekst zoals deze werd aangeleverd. Dit Nederlandstalig abstract onderging geen peer review. Het redactioneel proces van The Lancet werd enkel uitgevoerd op de originele Engelse tekst, welke geldt als de referentie voor dit artikel.

Supplement to: Horváth-Puhó E, van Kassel MN, Gonçalves BP, et al. Mortality, neurodevelopmental impairments, and economic outcomes after invasive group B streptococcal disease in early infancy in Denmark and the Netherlands: a national matched cohort study. *Lancet Child Adolesc Health* 2021; published online April 21. http://dx.doi.org/10.1016/S2352-4642(21)00022-5.

Samenvatting

Achtergrond Groep B *streptokokken* (GBS) ziekte is een veel voorkomende oorzaak van neonatale sterfte. De uitkomsten op de lange termijn zijn nauwelijks onderzocht. Het doel van deze studie was om inzicht te krijgen in sterfte, neurologische schade en economische gevolgen op de lange termijn in Deense en Nederlandse kinderen die invasieve GBS-ziekte in de eerste drie maanden van het leven hebben doorgemaakt.

Methode: Deense en Nederlandse kinderen die in het verleden GBS-ziekte als baby hadden doorgemaakt werden opgezocht in bestaande nationale databestanden van ziekenhuizen en microbiologische referentie laboratoria. GBS blootgestelde kinderen werden gedefinieerd als kinderen die een invasieve GBS-ziekte (sepsis, meningitis of pneumonie) hadden doorgemaakt tot de leeftijd van 89 dagen. Voor elk blootgesteld kind werden er tien niet-blootgestelde kinderen van hetzelfde geslacht, geboorte jaar/maand en zwangerschapsduur random geselecteerd. Sterfte werd geanalyseerd door middel van *Cox proportional hazards* modellen. De neurologische ontwikkelingsuitkomst werd bepaald op basis van ontslagdiagnoses uit nationale patiëntenregistraties (Denemarken) en speciaal onderwijsregistraties (Nederland). Ook werden de uitkomstmaten "gebruik van gezondheidszorg" en "inkomen per huishouden" vergeleken tussen de blootgestelde en niet-blootgestelde kinderen.

Resultaten: 2258 kinderen (1561 Deense kinderen geboren tussen 1 januari 1997 en 31 december 2017 en 697 Nederlandse kinderen geboren tussen 1 januari 2000 en 31 december 2017) met invasieve GBS-ziekte werden gevonden. De uitkomsten werden na gemiddeld 14 jaar (IQR 7-18 jaar) in Denemarken en 9 jaar (IQR 9-11 jaar) in Nederland bepaald. 366 kinderen hadden een GBS-meningitis, 1763 hadden een GBS-sepsis en 129 hadden een GBS-pneumonie doorgemaakt. Deze blootgestelde kinderen werden gematcht aan 22462 niet-blootgestelde kinderen. GBS meningitis was geassocieerd met een verhoogde sterfte op de leeftijd van 5 jaar (*adjusted hazard ratio:* 4·08 [95% CI 1·78–9·35] in Denemarken en 6·73 [3·76–12·06] in Nederland). Zowel GBS-sepsis als GBS-meningitis was geassocieerd met een verhoogd risico op neurologische schade op de leeftijd van 10 jaar (*risk ratio*: 1·77 [95% CI 1·44–2·18]) in Denemarken en 2·28 [1·64–3·17] in Nederland). Het doormaken van GBS-ziekte leidt ook tot meer bezoek aan de polikliniek (*incidence rate ratio* 1·93 [95% CI 1·79–2·09], $p<0{\cdot}0001$) en ziekenhuis opnames (1·33 [1·27–1·38], $p<0{\cdot}0001$) in kinderen tot de leeftijd van 5 jaar. Er werd geen verschil tussen het huishoudelijk inkomen van blootgestelde en niet-blootgestelde kinderen gevonden.

Interpretatie: Het doormaken van invasieve GBS-ziekte, met name meningitis, in de eerste levensmaanden is geassocieerd met hogere sterfte en een hoger risico op neurologische schade op de kinderleeftijd. Deze tot nu toe onbekende uitkomst draagt bij aan de ziektelast van GBS-ziekte en onderstreept het belang van de ontwikkeling van preventie door middel van maternale GBS-vaccinatie. Daarnaast is het belangrijk om de neurologische ontwikkeling van kinderen die GBS-ziekte hebben doorgemaakt op de lange termijn te volgen.
