# Supplementary material for: Mortality, neurodevelopmental impairments, and economic outcomes after invasive group B streptococcal disease in early infancy in Denmark and the Netherlands: a national matched cohort study
Source: Lancet Child Adolesc Health. 2021 Jun;5(6):398–407. doi: 10.1016/S2352-4642(21)00022-5 (PMC8131199; doi:10.1016/S2352-4642(21)00022-5)
Supplement: Danish translation of the abstract [file mmc2.pdf]

THE LANCET
Child & Adolescent Health

Supplementary appendix 2

This translation in Danish was submitted by the authors and we reproduce it as supplied. It has not been peer reviewed. *The Lancet*'s editorial processes have only been applied to the original in English, which should serve as reference for this manuscript.

Denne danske oversættelse er udarbejdet af forfatterne og vi gengiver den som leveret. Det danske resumé er ikke peer-reviewed. Kun den engelske originaltekst har gennemgået The Lancets redaktionelle processer og det er således den engelske tekst, som er gældende som reference for denne artikel.

Supplement to: Horváth-Puhó E, van Kassel MN, Gonçalves BP, et al. Mortality, neurodevelopmental impairments, and economic outcomes after invasive group B streptococcal disease in early infancy in Denmark and the Netherlands: a national matched cohort study. *Lancet Child Adolesc Health* 2021; published online April 21. http://dx.doi.org/10.1016/S2352-4642(21)00022-5.

Resumé

Baggrund
Gruppe B *streptokok* (GBS)-sygdom er en årsag til neonatal død, men dens langsigtede virkninger efter infektion i den tidlige barndom er ikke undersøgt. Formålet med dette studie var at undersøge dødelighed, neurologiske udviklingsforstyrrelser og økonomiske følger efter invasiv GBS (iGBS)-sygdom i spædbarnsalderen og frem til ungdomsårene i Danmark og Holland.

Metode
I dette kohortestudie har vi identificeret danske og hollandske børn med iGBS-sygdom ved hjælp af nationale sundhedsregistre, administrative databaser og laboratoriedatabaser som bekræftede børnenes diagnoser. Børn blev defineret som eksponerede hvis de havde haft iGBS-sygdom (sepsis, meningitis eller lungebetændelse) inden for 89 dage efter de blev født. For hvert eksponeret barn blev 10 ikke-eksponerede børn tilfældigt udvalgt og matchet efter køn, fødselsår og -måned og svangerskabsalder. Data om dødelighed blev analyseret ved hjælp af *Cox proportional hazards*-modeller. Data om neurologiske udviklingsforstyrrelser frem til ungdomsårene blev indhentet fra udskrivningsdiagnoser i Landspatientregisteret (Danmark) og fra særlige undervisningsregistre (Holland). Brug af sundhedsydelser og husstandsindkomst blev også sammenlignet blandt de eksponerede og ikke-eksponerede kohorter.

Resultater
Vi identificerede 2.258 børn – 1.561 født i Danmark i perioden 1. januar 1997 til 31. december 2017 og 697 født i Holland i perioden 1. januar 2000 til 31. december 2017 – med iGBS-sygdom. Median follow-up-tid var henholdsvis 14 år (IQR 7-18) i Danmark og 9 år (IQR 6-11) i Holland. 366 børn havde meningitis, 1.763 havde sepsis og 129 havde lungebetændelse (kun i Danmark). Disse børn blev matchet med 22.462 børn som ikke havde haft iGBS-sygdom. iGBS-meningitis var forbundet med en øget dødelighed ved 5-års alderen (justeret *hazard ratio* 4,08 [95 % konfidensinterval 1,78–9,35] for Danmark og 6,73 [3,76–12,06] for Holland). Alle iGBS-sygdomme var forbundet med en øget risiko for neurologiske udviklingsforstyrrelser ved 10-års alderen, både i Danmark (relativ risiko 1,77 [95 % konfidensinterval 1,44–2,18]) og i Holland (2,28 [1,64–3,17]). En iGBS-diagnose var forbundet med hyppigere ambulante behandlinger (incidensrateratio 1,93 [95 % konfidensinterval 1,79–2,09], p<0,0001) og hospitalsindlæggelser (1,33 [1,27–1,38], p<0·0001) hos børn på 5 år eller yngre. Vi fandt ikke nogle forskelle i husstandsindkomst blandt de eksponerede og ikke-eksponerede kohorter.

Konklusion
iGBS-sygdom, særligt meningitis, var forbundet med en øget mortalitet og en højere risiko for neurologisk udviklingsforstyrrelser senere i barndommen. Denne byrde, som ikke tidligere er kvantificeret, understreger vigtigheden af en GBS-vaccine samt behovet for at følge og behandle personer, som har haft og er overlevet iGBS-sygdom.
