# Supplementary material for: Mortality, neurodevelopmental impairments, and economic outcomes after invasive group B streptococcal disease in early infancy in Denmark and the Netherlands: a national matched cohort study
Source: Lancet Child Adolesc Health. 2021 Jun;5(6):398–407. doi: 10.1016/S2352-4642(21)00022-5 (PMC8131199; doi:10.1016/S2352-4642(21)00022-5)
Supplement: Supplementary appendix 3 [file mmc3.pdf]

THE LANCET
Child & Adolescent Health

Supplementary appendix 3

This appendix formed part of the original submission and has been peer reviewed. We post it as supplied by the authors.

Supplement to: Horváth-Puhó E, van Kassel MN, Gonçalves BP, et al. Mortality, neurodevelopmental impairments, and economic outcomes after invasive group B streptococcal disease in early infancy in Denmark and the Netherlands: a national matched cohort study. *Lancet Child Adolesc Health* 2021; published online April 21. http://dx.doi.org/10.1016/S2352-4642(21)00022-5.

Invasive Group B streptococcal disease in early infancy in Denmark and the Netherlands: National matched cohort study of mortality, neurodevelopmental impairments, and economic outcomes

Erzsébet Horváth-Puhó PhD*[1], Merel N. van Kassel MD*[2], Bronner P. Gonçalves MD*[3,4], Brechje de Gier PhD[5], Simon R. Procter DPhil[3,4], Proma Paul DrPH[3,4], Arie van der Ende PhD[6], Kirstine Kobberøe Søgaard PhD[1,7], Susan J.M. Hahné PhD[5], Jaya Chandna MPhil[3,4], Stephanie J. Schrag DPhil[8], Diederik van de Beek PhD[2], Mark Jit PhD[#3,4], Henrik Toft Sørensen MD[#1], Merijn W. Bijlsma PhD[#2,9], Joy E Lawn PhD[#3,4]

[1] Department of Clinical Epidemiology, Aarhus University, Olof Palmes Allé 43-45, 8200, Aarhus N, Denmark

[2] Amsterdam UMC, University of Amsterdam, Department of Neurology, Amsterdam Neuroscience, Meibergdreef 9, 1100 DD Amsterdam, The Netherlands

[3] Maternal, Adolescent, Reproductive & Child Health (MARCH) Centre, London School of Hygiene & Tropical Medicine, Keppel Street, London, WC1E 7HT, UK

[4] Department of Infectious Disease Epidemiology, London School of Hygiene & Tropical Medicine, Keppel Street, London, WC1E 7HT, UK.

[5] Centre for Infectious Disease Control, National Institute for Public Health and the Environment, Antonie van Leeuwenhoeklaan 9, 3721 MA Bilthoven, The Netherlands

[6] Netherlands Reference Laboratory for Bacterial Meningitis, Amsterdam UMC/RIVM, University of Amsterdam, Department of Medical Microbiology and Infection Prevention, Amsterdam Infection and Immunity, Meibergdreef 9, 1100 DD Amsterdam, The Netherlands

[7] Department of Clinical Microbiology, Aalborg University Hospital, Hobrovej 18-22, 9000 Aalborg, Denmark

[8] Division of Bacterial Diseases, Centers for Disease Control and Prevention, 1600 Clifton Rd, MS C25, 30329, Atlanta, GA, USA

[9] Amsterdam UMC, University of Amsterdam, Department of Pediatrics, Meibergdreef 9, 1100 DD Amsterdam, The Netherlands

*Joint first authors: Erzsébet Horváth-Puhó[1], Merel N. van Kassel[2], Bronner P. Gonçalves[3,4]
# Joint senior authors: Mark Jit PhD[3,4], Henrik Toft Sørensen[1], Merijn W. Bijlsma[2,9], Joy E. Lawn[3,4]

Contents

STROBE checklist (page 3)

Supplementary Methods (pages 5 – 9)
Additional information on databases (Denmark)
Additional information on databases (the Netherlands)
Linkage procedure among the different databases in the Netherlands

Supplementary Tables (pages 10 – 23)
Supplementary Table S1 - Health registers and variables used in the study
Supplementary Table S2 – Sensitivity analysis: mortality rates and hazard ratios for comparisons between exposed and unexposed groups not matched by gestational age
Supplementary Table S3 - NDI outcomes in terms of any domain or domain-specific need, or special education need, by iGBS clinical syndrome.
Supplementary Table S4 - Special education requirements by affected domain
Supplementary Table S5 - Potential study biases in the assessments of exposure and outcome

Supplementary Figures (pages 21 - 24)
Supplementary Figure S1 - Kaplan-Meier survival curves for children with invasive GBS disease compared to gestational age-matched unexposed children
Supplementary Figure S2 - Household income in families of exposed and unexposed children
Supplementary Figure S3 - Welfare received by households with exposed vs. unexposed children in the Netherlands

References (page 25)

STROBE checklist.

Our study is reported according to the Strengthening the Reporting of Observational Studies in Epidemiology (STROBE) statement. The STROBE checklist, downloaded from https://www.strobe-statement.org, is shown below. For items 7 and 8 (related to diagnostic criteria and comparability of assessment methods) relevant information is included both in the *Methods* section of the main text and in **Supplementary Table S1**.

	**Item**	**Recommendation**	**Reported on manuscript page**
**Title and abstract**	1	(*a*) Indicate the study's design with a commonly used term in the title or the abstract	1
		(*b*) Provide in the abstract an informative and balanced summary of what was done and what was found	3
**Introduction**			
Background/ rationale	2	Explain the scientific background and rationale for the investigation being reported	4
Objectives	3	State specific objectives, including any prespecified hypotheses	4
**Methods**			
Study design	4	Present key elements of study design early in the paper	5
Setting	5	Describe the setting, locations, and relevant dates, including periods of recruitment, exposure, follow-up, and data collection	5-6, supplementary
Participants	6	(*a*) Give the eligibility criteria, and the sources and methods of selection of participants. Describe methods of follow-up	5-6, supplementary
		(*b*) For matched studies, give matching criteria and number of exposed and unexposed	5-6, supplementary
Variables	7	Clearly define all outcomes, exposures, predictors, potential confounders, and effect modifiers. Give diagnostic criteria, if applicable	6-7, supplementary
Data sources/ measurement	8	For each variable of interest, give sources of data and details of methods of assessment (measurement). Describe comparability of assessment methods if there is more than one group	5-7, supplementary
Bias	9	Describe any efforts to address potential sources of bias	5-7, supplementary
Study size	10	Explain how the study size was arrived at	5-6, supplementary
Quantitative variables	11	Explain how quantitative variables were handled in the analyses. If applicable, describe which groupings were chosen and why	5-7
Statistical methods	12	(*a*) Describe all statistical methods, including those used to control for confounding	8
		(*b*) Describe any methods used to examine subgroups and interactions	8
		(*c*) Explain how missing data were addressed	8
		(*d*) If applicable, explain how loss to follow-up was addressed	8, supplementary
		(*e*) Describe any sensitivity analyses	9
**Results**			
Participants	13	(a) Report numbers of individuals at each stage of study—eg numbers potentially eligible, examined for eligibility, confirmed eligible, included in the study, completing follow-up, and analysed	9, figure 1
		(b) Give reasons for non-participation at each stage	9, figure 1

		(c) Consider use of a flow diagram	Figure 1
Descriptive data	14	(a) Give characteristics of study participants (eg demographic, clinical, social) and information on exposures and potential confounders	9, table 1
		(b) Indicate number of participants with missing data for each variable of interest	9, figure 1
		(c) Summarise follow-up time (eg, average and total amount)	9
Outcome data	15	Report numbers of outcome events or summary measures over time	9-11, figure 1
Main results	16	(*a*) Give unadjusted estimates and, if applicable, confounder-adjusted estimates and their precision (eg, 95% confidence interval). Make clear which confounders were adjusted for and why they were included	9-11, tables, supplementary
		(*b*) Report category boundaries when continuous variables were categorized	9-11, tables, supplementary
		(*c*) If relevant, consider translating estimates of relative risk into absolute risk for a meaningful time period	9-11, tables
Other analyses	17	Report other analyses done—eg analyses of subgroups and interactions, and sensitivity analyses	9-11, tables, supplementary
**Discussion**			
Key results	18	Summarise key results with reference to study objectives	11-14
Limitations	19	Discuss limitations of the study, taking into account sources of potential bias or imprecision. Discuss both direction and magnitude of any potential bias	14
Interpretation	20	Give a cautious overall interpretation of results considering objectives, limitations, multiplicity of analyses, results from similar studies, and other relevant evidence	11-14, supplementary
Generalisability	21	Discuss the generalisability (external validity) of the study results	14
**Other information**			
Funding	22	Give the source of funding and the role of the funders for the present study and, if applicable, for the original study on which the present article is based	9&16

Supplementary Methods

Additional information on national databases

In this section of the Supplementary Appendix, we provide additional information on the national databases that were used both to assess exposure (history of invasive GBS disease [iGBS]) and outcomes (mortality, neurodevelopment impairments, household income, and healthcare utilization).

Note: In the two countries where this study was performed, a risk-based intrapartum antibiotic prophylaxis policy was adopted nearly two decades ago. However, data are not available on use of intrapartum antibiotic prophylaxis for individual infants.

Denmark
The Danish National Health Service provides tax-supported health care, ensuring unfettered access to general practitioners and hospitals for all Danish inhabitants.[1] Accurate linkage of all registries is possible in Denmark at the individual level using the unique Civil Personal Register (CPR) number assigned to each Danish citizen at birth and to residents upon immigration.[2]

In our analyses, the following databases were used:

- Danish Civil Registration System: This database is an administrative register established in 1968. It contains individual-level information on all persons residing in Denmark. Civil Registration System provides daily updates on vital statistics, including dates of birth, migration, emigration, and death. Upon registration in the Civil Registration System, each person receives a unique ten-digit identification number (CPR number), which allows for cost-effective and unambiguous individual-level record linkage of different Danish registers. The CPR number is used in all Danish administrative and medical registers containing birth-related, vital status, clinical, healthcare utilization, and income data.[2]

- Danish Medical Birth Registry: This is a key component of the Danish health information system. The Medical Birth Registry permits the health of pregnant women and their offspring to be monitored. The register was established in 1973 based on paper birth forms and includes prospectively collected data on all deliveries in Denmark. Major changes in the construction and content of the Medical Birth Registry were implemented in 1997 when the electronic registration of births replaced paper forms. The Medical Birth Registry contains information on the index pregnancy (including CPR numbers of the parents), pregnancy-related characteristics of the mother (e.g. parity, pregnancy-related complications), details of the delivery (e.g. date of delivery, caesarean section), and outcome characteristics of the newborn (e.g. gestational age, Apgar score, birth weight).[3]

- Danish National Patient Registry: This registry is one of the world's oldest nationwide hospital registries and is used extensively for research.[4-6] The National Patient Registry contains information recorded on all admissions to Danish non-psychiatric hospitals since 1977 and on outpatient clinic visits and emergency room visits since 1995. Each hospital discharge or outpatient clinic visit is recorded in the National Patient Registry with one primary diagnosis and one or more secondary diagnoses classified according to the International Classification of Diseases, Eighth Revision (ICD-8) through 1993 and Tenth Revision (ICD-10) thereafter. The National Patient Registry contains information also on examinations, certain inpatient medical treatments, and surgical procedures.[7]

- Danish Psychiatric Central Research Registry: This database contains information on every psychiatric admission from 1969 until the present. In 1995, data on outpatient clinic treatment and emergency room contacts were added and the Psychiatric Central Research Registry became an integrated component of the National Patient Registry.[8] The register contains the CPR-number, dates of any admission and discharge or start and end of any outpatient treatment including emergency room visits; all diagnoses; type of referral; place of treatment with identification of the specific department; municipality of residence; and mode of admission (acute or planned).

- Income Statistics Register: Provided by Statistics Denmark, this dataset contains high-quality data on the income composition of the Danish population, including variables related to individual income (e.g. salary, income from private pensions). The data are available from 1980 until the present.[9] Gross income was measured before deductions of labor market and special pension contributions. Income values are available in Danish Krone in the Income Statistics Register. These income values, used in our study, were converted from Krone to Euros.[10,11] Annual household incomes were defined as the sum of the gross income of the cohort member's parents.

Linkage procedure among the different databases in Denmark

Below we describe the steps used in linkage of databases. Accurate linkage of the used medical and administrative registries was possible in Denmark at the individual level using the unique CPR number.

- Step 1. 1,297,383 live births were identified from the Medical Birth Registry in Denmark in the study period (from January 1, 1997 to December 31, 2017). These data were linked to the National Patient Registry to identify all children with a diagnosis of invasive GBS disease in the first 90 days after birth. GBS meningitis, sepsis, and pneumonia were defined based on discharge diagnoses using ICD-10 codes (sepsis: P36.0, A40.1; meningitis: G00.2, [P36.0 or A40.1] and [G00.9 or G03.9]; pneumonia: P23.3, J15.3). Both, primary and secondary discharge diagnoses were included. We identified 1,561 children with a history of invasive GBS disease.

- Step 2. In the primary analysis, a matched comparison cohort of children without invasive GBS disease was created by linking the Medical Birth Registry to the Civil Registration System. For each child with a history of invasive GBS disease, up to 10 infants were randomly selected from the Medical Birth Registry, and were matched on sex, year/month of birth, and gestational age categories (<28 weeks [extreme preterm], 28-36 weeks [preterm], and ≥37 weeks [term]). We used matching without replacement. Two percent of the children with iGBS had fewer than 10 matched counterparts. Data on gestational age was missing for 28 neonates, therefore they were not included in the primary analyses. The children's birth dates were defined as the index dates for members of the GBS cohort and their matched counterparts in the comparison cohort. We included 15,501 unexposed children in the primary analyses.

- Step 3. In addition to our primary comparison cohort, which was matched on gestational age, we also compared the children with a history of iGBS to a second unexposed cohort matched on sex and birth month/year but not on gestational age. The children with missing gestational age were included in these analyses. In this sensitivity analysis, we used the database linkage methods described in Step 2.

All linkage, data management steps, and statistical analyses were performed using SAS version 9.4 (SAS Institute, Cary, NC).

The Netherlands

The Dutch Healthcare System is a social welfare-based system, with mandatory health care insurance for all residents aged 18 years or older. The System provides a standard, nearly comprehensive, benefit package specified by law.[12]

The following databases were used in this cohort study:

- The Netherlands Reference Laboratory for Bacterial Meningitis database: The Reference Laboratory started collecting *Neisseria meningitides* isolates in 1959 and of other bacteria causing meningitis from 1975 onwards, covering approximately 90 percent of all isolates cultured from CSF of patients with a (suspected) bacterial meningitis in the Netherlands.[13,14] Additionally, microbiology laboratories in the Netherlands also send blood isolates of infants with invasive GBS infection without a suspicion of bacterial meningitis. A minimal set of patient characteristics are available for these patients including date of birth, sex, residency, and date that the culture was performed. The first reported date of illness, mostly the first date a culture was taken, was used to calculate age of onset. If not reported (2.6% of patients), the date the isolate was sent to or received by the Reference Laboratory was used.

- PeriNed: The Perinatal Registry has covered approximately 99% of all births in the Netherlands since 2000.[15-17] It contains data provided by four groups of professionals (e.g. midwives, general practitioners, gynecologists and pediatricians/neonatologists) involved in childbirth care. This data includes variables related to pregnancy, delivery and neonatal (re)admission up until 28 days of age. The registry is managed by PeriNed on behalf of four professional associations: the KNOV (Royal Dutch Organization of Obstetricians), the LHV (National General Practitioners Association), the NVOG (Dutch Association for Obstetrics and Gynecology) and the NVK (Dutch Association for Pediatrics).

- Statistics Netherlands: Statistics Netherlands provides datasets with individual level data to researchers to conduct their own research under strict conditions (For further information: microdata@cbs.nl). Population registry datasets containing variables related to health, well-being, income, and education are linkable using a unique central personal registry number, which is assigned to each Dutch citizen at birth and to residents upon immigration. Population and time periods differ among datasets. All of the results presented in this paper are based on calculations by researchers of Amsterdam UMC and RIVM using the non-public datasets from Statistics Netherlands.

- Dutch Hospital data registry: There are three types of non-public registries available from Statistics Netherlands: hospital admission data (including date of admission and discharge, main diagnosis associated with the hospital admission), diagnosis data (including all diagnoses registered during admission) and data on all care/procedures registered during admission. In the current study, only permission to use these datasets for linkage of patients (see linkage procedure below) was obtained, so these datasets were not used for further analyses. Datasets are available from 1995 to 2012.

- Municipal Personal Records Database: This is a national database recording deaths and date of deaths in the Netherlands. The dataset contains information from 1995 to 2019, and is updated every year.

- Dutch Income Panel Survey*:* This is a national database describing household income for every Dutch household on January 1st of each year, covering data from 2003 to 2018. Starting at age 1, children can be linked to their respective households using a linkage table of individuals and households. For this reason, our analyses of household income started at age 1. From the same datasets, the percentage of gross household income from welfare payments were available for 2011-2018. The term "welfare" is defined broadly and includes all social benefits such as income-dependent assistance to help pay for rent or health care. This results in a very high percentage of households receiving welfare. The two outcomes examined in this study were proportion of households receiving welfare payments and annual amount of welfare payments to qualifying households.

- Dutch Primary School Registry and Dutch Special Education School Registry: This national database on primary school registration and special education was used to identify children in the exposed and unexposed groups who required either enrollment in special schools or additional support in regular schools. Its data cover the period 2008-2019. In the current study, registration in special schools was considered evidence of severe impairment and additional support in standard schools was considered evidence of moderate impairment. To handle missing data in the Netherlands for children who began to be followed in the education database at an older age due to data availability (for example, for a child who was already 8 years old in 2008), we assumed that the outcome at that older age was the same as during preceding years.

Linkage procedure among the different databases in the Netherlands

Below we describe the series of steps used in linkage of databases:

- Step 1. All infants aged less than 90 days with a CSF and/or blood culture positive for GBS received by the Netherlands Reference Laboratory for Bacterial Meningitis between January 1, 1987 and December 31, 2017 were identified. This resulted in 1591 episodes in 1578 patients.
- Step 2. This dataset was transferred to the secure environment of Statistics Netherlands and matched to unique central personal registry numbers based on the date of birth, sex and residency. For 568 (36%) infants, there was a single match between an infant in the Reference Laboratory dataset and a personal registry number in

Statistics Netherlands. For 806 (51%) infants, there were multiple personal registry number options within Statistics Netherlands that could be matched to the infant in the Reference Laboratory dataset, due to multiple infants with the same date of birth, sex and residency. For 202 (13%) infants in the Reference Laboratory, no personal registry number could be linked, mostly due to missing values for place of residency and/or sex.

- Step 3. In order to link the correct personal registry number in Statistics Netherlands to infants included in the Reference Laboratory, the datasets of PeriNed and the Dutch Hospital Data Registry were accessed. All remaining infants in the Reference Laboratory (n=1376) were manually checked against potential personal registry numbers in both datasets:

    - Step 3a. All personal registry numbers in PeriNed, with a plausible admission date and duration; a diagnosis of "invasive GBS disease (n=484)" or "sepsis/meningitis (n=55)" or "infection (n=8)"; or with an antibiotic treatment duration of >3 days (n=9) were considered to indicate the correct patients.
    - Step 3b. All personal registry numbers in the Dutch Hospital Data Registry associated with a plausible admission date and duration; a primary diagnosis of "streptococcal sepsis/meningitis or infection (n=233)"; or a sub-diagnosis of "streptococcal sepsis/meningitis or infection (n=34)" were considered to indicate the correct patients.

- Step 4. 528 infants in the Reference Laboratory dataset matched with a unique personal registry number in PeriNed, 167 matched with a unique personal registry number in the Dutch Hospital Data Registry, and 28 matched in both. Of the remaining 723 infants in the Reference Laboratory, 232 belonged to the group that was matched with a single personal registry number in Statistics Netherlands (Step 2). These 232 personal registry numbers were assumed to be linked correctly, and the infants were included in our analyses.

- Step 5. The two cohorts described in the manuscript were generated as follows:
    - Cohort matched by gestational age (primary analysis): since the variable 'gestational age' was provided by the PeriNed registry, only children born during 2000-2017 could be included in this cohort. Of the 1,043 patients born during 2000-2017 with a history of iGBS (Reference Laboratory), 734 were matched with a personal registry number in Statistics Netherlands. Merging these cases with the PeriNed database resulted in 697 matched patients and 324 patients that could not be found in the PeriNed registry (Figure 1, main manuscript). In the Netherlands, 0.3% of the children with iGBS had fewer than 10 matched counterparts.

    Note: In Step 3a the PeriNed database was used to link patients in the Reference Laboratory to a personal registry number based on diagnoses/antibiotic treatment/duration of admission. In Step 5, the linked patients were merged with the PeriNed registry for the 2000-2017 period. It is possible that we were not able to identify the right personal registry number in Step 3a since PeriNed is a birth registry covering birth up to the first month of life. For example, we may not have identified the correct personal registry number for an infant with GBS disease at 2 months of age (Step 3a), but it is likely that this infant still was registered at birth in the PeriNed registry and therefore correctly merged on personal registry number (found in Step 3b) in Step 5.

    - Cohort not matched by gestational age (sensitivity analysis): All patients linked to a personal registry number in Statistics Netherlands were used for this cohort. However, only 947 of the 954 cases could be retrieved in the Municipal Personal Records Database. Therefore 7 patients were deemed lost to follow up and excluded from the cohort.

All linkage, data management steps, and statistical analyses were performed using SPSS version 25 and STATA version 16.

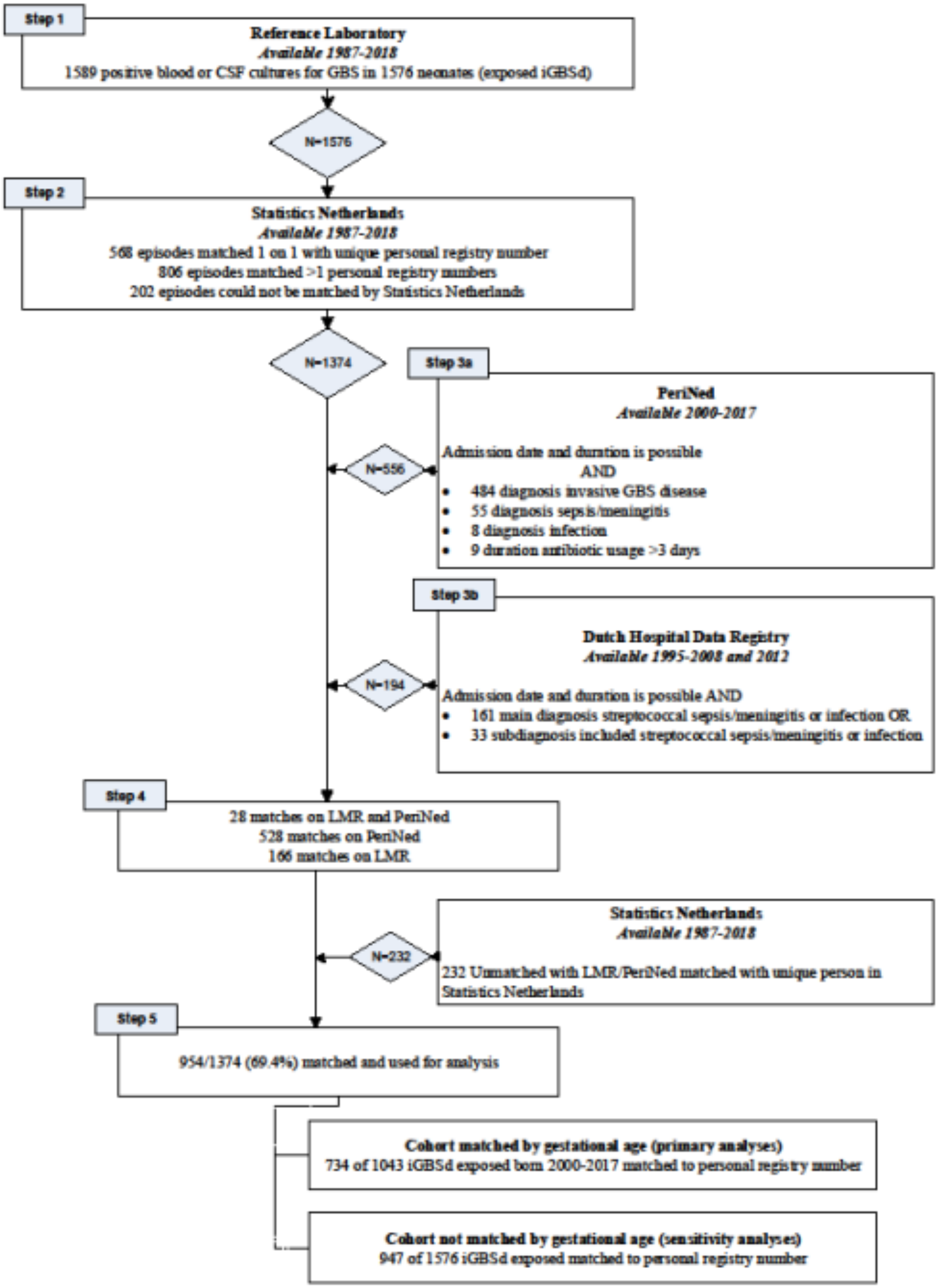
Step 1
Reference Laboratory
Available 1987-2018
1589 positive blood or CSF cultures for GBS in 1576 neonates (exposed iGBSd)
N=1576
Step 2
Statistics Netherlands
Available 1987-2018
568 episodes matched 1 on 1 with unique personal registry number
806 episodes matched >1 personal registry numbers
202 episodes could not be matched by Statistics Netherlands
N=1374
Step 3a
PeriNed
Available 2000-2017
Admission date and duration is possible
AND
484 diagnosis invasive GBS disease
55 diagnosis sepsis/meningitis
8 diagnosis infection
9 duration antibiotic usage >3 days
N=556
Step 3b
Dutch Hospital Data Registry
Available 1995-2008 and 2012
Admission date and duration is possible AND
161 main diagnosis streptococcal sepsis/meningitis or infection OR
33 subdiagnosis included streptococcal sepsis/meningitis or infection
N=194
Step 4
28 matches on LMR and PeriNed
528 matches on PeriNed
166 matches on LMR
Statistics Netherlands
Available 1987-2018
232 Unmatched with LMR/PeriNed matched with unique person in Statistics Netherlands
N=232
Step 5
954/1374 (69.4%) matched and used for analysis
Cohort matched by gestational age (primary analyses)
734 of 1043 iGBSd exposed born 2000-2017 matched to personal registry number
Cohort not matched by gestational age (sensitivity analyses)
947 of 1576 iGBSd exposed matched to personal registry number

Supplementary Tables

Table S1. Health Registers and variables used in the study.

	Denmark	The Netherlands
**Exposures**		
**GBS sepsis**	**Danish National Patient Registry**<br>*(Including in- and outpatients, primary and secondary discharge diagnoses)*	**Netherlands Reference Laboratory for Bacterial Meningitis**
	ICD-10 codes ≤ 89 days after birth:<br>· P36.0 (Sepsis of newborn due to GBS)<br>· A40.1 (Sepsis due to GBS)	Positive blood culture for GBS
**GBS meningitis**	**Danish National Patient Registry**<br>*(Including in- and outpatients, primary and secondary discharge diagnoses)*	**Netherlands Reference Laboratory for Bacterial Meningitis**
	ICD-10 codes ≤ 89 days after birth:<br>· G00.2 (Streptococcal meningitis)<br>· Patients with:<br>{[P36.0 (Sepsis of newborn due to GBS) or<br>A40.1 (Sepsis due to GBS)]<br>**AND**<br>[G00.9 (Bacterial meningitis, unspecified) or<br>G03.9 (Meningitis, unspecified)]}	Positive cerebrospinal fluid (CSF) **OR** CSF and blood culture positive for GBS
**GBS pneumonia**	**Danish National Patient Registry**<br>*(Including in- and outpatients, primary and secondary discharge diagnoses)*	-
	ICD-10 codes ≤ 89 days after birth:<br>· P23.3 (Congenital pneumonia due to GBS)<br>· J15.3 (Pneumonia due to GBS)	NA

Descriptive characteristics

**Sex**	**Danish Civil Registration System**	**Netherlands Reference Laboratory for Bacterial Meningitis**
**Gestational age**	**Danish Medical Birth Registry**	**PeriNed**
	Classification<br>· Extreme preterm: <28 weeks<br>· Preterm: 28-36 weeks<br>· Term: ≥37 weeks	
**Birth weight**	**Danish Medical Birth Registry**	**PeriNed**
	In grams	
**Multiplicity**	**Danish Medical Birth Registry**	**PeriNed**
	Singletons, twins, higher order	
**Maternal age**	**Danish Medical Birth Registry**	**PeriNed**
	Maternal age on the date of child´s birth	

Outcomes

**Mortality**<br>**(all-cause mortality)**	**Danish Civil Registration System**<br>(*updated daily*)	**Statistics Netherlands:**<br>· **Municipal Personal Records Database**<br>(*Available 1995-2019; updated yearly*)

	Classification<br>· Death during index hospitalisation<br>· Death during acute phase: died <90 days of age<br>· 5-years mortality: died ≤ 5 years of age	Classification<br>· Death during acute phase: died <90 days of age<br>· 5-years mortality: died ≤ 5 years of age
**Neurodevelopment impairment**	**Danish National Patient Registry and Danish Psychiatric Central Research Register**<br>*(Including inpatient and outpatients, primary and secondary discharge diagnoses*)	**Statistics Netherlands:**<br>· **Dutch Primary School Registry** *(available 2008-2019*)<br>· **Dutch Special Education School Registry** *(available 2008-2019*)
Any	Any of the domain specific ICD-10 codes listed below:<br>· Mild: One or two domain-specific mild codes<br>· Moderate: Three or more mild codes **OR** one moderate code<br>· Severe: Two or more moderate codes **OR** at least one severe code	Based on labels in registry files<br>· Mild: Label for special education need in Dutch Primary School Registry = attending regular school with additional support<br>· Moderate-Severe: Label for special education in Dutch Special Education School Registry = attending special school
Motor	Any of the ICD-10 codes listed below:<br>· Mild: F82, R27.0, R27.8, R26.0, R26.1, G24.9, G25.9 (developmental disorder of motor function, ataxia, other lack of coordination, ataxic or paralytic gait, dystonia, extrapyramidal and movement disorder)<br>· Moderate: G80.1, G80.3, G80.4, G80.8, G80.9 (spastic diplegic cerebral palsy, dyskinetic, ataxic or other cerebral palsy)<br>· Severe: G80.0 (Spastic quadriplegic cerebral palsy)	Label(s): physical impairment
Hearing	Any of the ICD-10 codes listed below:<br>· Mild: H90.1, H90.4, H90.7 (unilateral hearing loss with unrestricted hearing on contralateral side [conductive and/or sensorineural])<br>· Severe: H90.0, H90.3, H90.6 (bilateral hearing loss [conductive and/or sensorineural])<br>· Not categorized: H90.2, H90.5, H90.8 (not specified hearing loss [conductive and/or sensorineural])	Label(s): hearing impaired, severe speech difficulties or deaf
Vision	Any of the ICD-10 codes listed below:<br>· Mild: H53.0, H53.1, H53.2, H53.4, H54.4, H54.5, H54.6 (amblyopia ex anopsia, subjective visual disturbances, diplopia, visual field	Label(s): visually impaired, visually handicapped or blind

	defects, blindness [monocular], moderate or severe visual impairment [monocular])<br>· Moderate: H54.2 (moderate visual impairment [binocular])<br>· Severe: H54.0, H54.1 (blindness [binocular], severe visual impairment [binocular])	
Cognitive	Any of the ICD-10 codes listed below:<br>· Mild: F70, F80.0, F80.1, F80.2, F80.9, F81.0, F81.1, F81.2 (mild mental retardation [MR], speech articulation disorder, expressive or receptive language disorder, other disorders of speech and language, reading or spelling disorder, disorder of arithmetic skills)<br>· Moderate: F71, F81.3, F83, F84.0, F84.1, F84.3, F84.5 (moderate MR, mixed disorder of scholastic skills, mixed developmental disorders, Autistic disorder, atypical autism, disintegrative disorder, Asperger syndrome)<br>· Severe: F72, F73, F80.3, F84.2, F84.4 (severe or profound MR, acquired aphasia with epilepsy, Rett's syndrome, overactive disorder with MR and stereotype movement)<br>· Not categorized: F78, F79, F84.8, F84.9, F88 (other MR, other pervasive developmental disorder, other disorders of psychological development)	Label(s): severe learning problems
Social/behavioral	Any of the ICD-10 codes listed below:<br><br>Mild: F90.0, F90.1, F90.2, F90.8, F90.9, F91.0, F91.1, F91.2, F91.3, F91.8, F91.9, F92.0, F92.8, F92.9, F93.0, F93.1, F93.2, F93.3, F93.8, F93.9, F94.1, F94.2, F94.8, F94.9, F95.1, F95.2, F95.8, F95.9, F98.0, F98.1, F98.2, F98.3 (attention-deficit hyperactivity disorders [predominantly inattentive, hyperactive, combined or other type], conduct disorder [confined to family context, childhood- or adolescent-onset type, other], oppositional defiant disorder, other conduct disorders, emotional disorders with onset specific to childhood, disorders of social functioning, Tic disorder, other behavioral and emotional disorders)	
Multi domain	If child has more than one affected domain AND<br>· Mild: Two domains mildly affected	

	· Moderate: Three or more domains mildly affected, **OR** one moderately affected and at least one mildly affected<br>· Severe: Two or more domains moderately affected, **OR** one domain severely affected and at least one domain mildly or moderately affected, OR two or more domains severely affected		

N.A.: not available for this country

Table S2. Sensitivity analysis: Mortality rates and hazard ratios for comparisons between exposed and unexposed groups not matched by gestational age.*

	Any invasive GBS			*GBS meningitis*			*GBS sepsis*		
	Exposed	Unexposed		Exposed	Unexposed		Exposed	Unexposed	
	Mortality Rate (95% CI)	Mortality Rate (95% CI)	HR (95% CI)	Mortality Rate (95% CI)	Mortality Rate (95% CI)	HR (95% CI)	Mortality Rate (95% CI)	Mortality Rate (95% CI)	HR (95% CI)
**Denmark**									
**0-89d**	97·5 (66·1-128·9)	13·7 (10·0-17·4)	7·83 (5·06-12·11)	171·8 (44·5-299·1)	11·9 (1·5-22·3)	17·65 (5·17-60·31)	87·9 (54·7-121·0)	14·1 (9·9-18·3)	7·10 (4·33-11·63)
**0-5y**	5·6 (3·9-7·4)	1·0 (0·8-1·3)	5·82 (3·94-8·59)	12·2 (4·2-20·2)	0·6 (0·1-1·2)	22·88 (7·04-74·29)	4·9 (3·1-6·7)	1·1 (0·8-1·3)	5·01 (3·21-7·83)
**The Netherlands**									
**0-89d**	299·9 (227·6-372·3)	16·6 (11·3-21·8)	17·82 (11·96-26·57)	339·2 (206·2-472·2)	19·5 (9·6-29·3)	17·24 (9·09-32·70)	280·1 (194·4-365·9)	15·1 (8·9-21·3)	18·25 (10·96-30·42)
**0-5y**	19·3 (14·9-23·8)	1·2 (0·9-1·6)	15·48 (10·74-22·30)	20·4 (12·7-28·2)	1·3 (0·7-1·9)	14·77 (8·21-26·48)	18·7 (13·3-24·2)	1·1 (0·7-1·5)	15·98 (10·02-25·48)

Abbreviations: HR: Hazard ratio, d: days, y: years

* Mortality rates are expressed as events per 1,000 child-years. Hazard ratios were adjusted for matching variables (i.e., sex and year of birth)

Table S3. **NDI outcomes in terms of any domain or domain-specific need, or special education need, by iGBS clinical syndrome.**

A. Denmark

	Any domain			*Domain specific*			*Multi domain*	
				**Cognitive**	**Motor**	**Social/Behavioral**		
	**Any**	**Mild**	**Moderate/ severe**	**Any**	**Any**	**Any**	**Any**	**Moderate/ severe**
**Age**	RR (95% CI)	RR (95% CI)	RR (95% CI)	RR (95% CI)	RR (95% CI)	RR (95% CI)	RR (95% CI)	RR (95% CI)
**<5y**	2·39 (1·82–3·14)	2·06 (1·30–3·26)	2·63 (1·85–3·73)	1·82 (1·15–2·89)	3·89 (2·53–5·97)	2·52 (1·54–4·12)	3·74 (2·05–6·83)	4·60 (2·49–8·51)
**<7y**	2·00 (1·58-2·54)	1·80 (1·24-2·60)	2·19 (1·59-3·02)	1·33 (0·89-1·97)	3·45 (2·24-5·31)	1·93 (1·33-2·79)	2·19 (1·33-3·63)	2·76 (1·62-4·68)
**<10y**	1·77 (1·44–2·18)	1·72 (1·28–2·31)	1·82 (1·33–2·49)	1·43 (1·03–2·00)	3·15 (1·98–5·02)	1·62 (1·22–2·15)	1·65 (1·05–2·60)	1·98 (1·22–3·21)
**<15y**	1·63 (1·33–2·00)	1·37 (0·99–1·89)	1·90 (1·43–2·53)	1·38 (1·02–1·87)	3·35 (2·03–5·52)	1·50 (1·14–1·97)	1·61 (1·07–2·42)	2·08 (1·36–3·19)
**<20y**	1·85 (1·34–2·54)	0·99 (0·52–1·87)	2·69 (1·81–4·00)	2·22 (1·48–3·32)	3·32 (1·42–7·75)	1·67 (1·06–2·64)	2·48 (1·36–4·51)	3·25 (1·74–6·05)

	GBS sepsis		*GBS meningitis*		*Early onset GBS*		*Late onset GBS*	
	**Any**	**Moderate/ severe**	**Any**	**Moderate/ severe**	**Any**	**Moderate/ severe**	**Any**	**Moderate/ severe**
**Age**	RR (95% CI)	RR (95% CI)	RR (95% CI)	RR (95% CI)	RR (95% CI)	RR (95% CI)	RR (95% CI)	RR (95% CI)
**<5y**	1·72 (1·22–2·43)	1·90 (1·21-2·97)	7·80 (4·42-13·77)	8·49 (4·28-16·86)	2·18 (1·60–2·97)	2·58 (1·75–3·80)	3·42 (1·88–6·20)	2·82 (1·24–6·41)
**<7y**	1·61 (1·21-2·15)	1·74 (1·17-2·57)	4·69 (2·78-7·89)	5·27 (2·80-9·92)	1·94 (1·49-2·52)	2·05 (1·43-2·95)	2·31 (1·32-4·04)	2·77 (1·40-5·46)
**<10y**	1·48 (1·15–1·90)	1·48 (1·00-2·20)	3·47 (2·19–5·50)	3·88 (2·15–6·99)	1·73 (1·37–2·17)	1·67 (1·17–2·38)	1·97 (1·21–3·20)	2·55 (1·32–4·93)
**<15y**	1·56 (1·23–1·97)	1·70 (1·22-2·38)	3·15 (1·82–5·46)	4·52 (2·35–8·67)	1·60 (1·28–2·00)	1·77 (1·30–2·42)	1·85 (1·07–3·20)	2·79 (1·42–5·48)
**<20y**	2·03 (1·44–2·87)	2·71 (1·77-4·15)	2·16 (0·52–9·02)	3·86 (0·81-18·40)	1·90 (1·36–2·64)	2·73 (1·80–4·14)	1·39 (0·43–4·49)	2·36 (0·68–8·24)

B. The Netherlands

	Any invasive GBS disease		*GBS sepsis*		*GBS meningitis*		*Early onset GBS*		*Late onset GBS*	
	**Any**	**Moderate/ severe**	**Any**	**Moderate/ severe**	**Any**	**Moderate/ severe**	**Any**	**Moderate/ severe**	**Any**	**Moderate/ severe**
**Age**	RR (95% CI)	RR (95% CI)	RR (95% CI)	RR (95% CI)	RR (95% CI)	RR (95% CI)	RR (95% CI)	RR (95% CI)	RR (95% CI)	RR (95% CI)
**<5y**	3·47 (2·06-5·82)	4·09 (2·27-7·37)	NA	NA	5·30 (2·57-10·89)	5·13 (2·24-11·79)	4·28 (2·19-8·39)*	5·89 (2·81-12·35)*	2·56 (1·14-5·77)	2·40 (0·89-6·51)
**<7y**	2·37 (1·57-3·56)	2·90 (1·83-4·62)	1·84 (1·06-3·18)	2·08 (1·09-3·98)	3·71 (1·05-6·72)	NA	2·89 (1·74-4·82)	3·61 (2·03-6·43)	1·69 (0·85-3·37)	2·05 (0·94-4·49)
**<10y**	2·28 (1·64-3·17)	2·70 (1·75-4·16)*	2·01 (1·30-3·01)	2·48 (1·38-4·47)*	2·81 (1·69-4·68)	3·05 (1·62-5·73)	2·54 (1·71-3·77)*	3·07 (1·82-5·16)*	1·85 (0·99-3·43)	2·09 (0·94-4·67)*
**<11y**	2·30 (1·63-3·23)	2·69 (1·70-4·26)*	1·88 (1·17-3·03)	2·25 (1·17-4·33)*	2·99 (1·83-4·88)	3·34 (1·77-6·33)	2·56 (1·69-3·87)*	2·98 (1·69-5·24)*	1·90 (1·02-3·52)	2·32 (1·03-5·22)

Abbreviations: NDI: neurodevelopmental impairment; iGBS: Invasive group B streptococcal disease; RR: Risk ratio

Categorisation of impairments by severity (mild, moderate, or severe) is specified in Table S1.

Risk ratio for the association between history of invasive GBS disease (all syndromes or syndrome specific) and NDI/education outcomes were estimated using a modified Poisson regression model.

* Due to the absence of cases in the extreme preterm gestational age category (<28 weeks), the preterm age categories were merged (<37 weeks) for adjustment purposes.

Table S4. Required special education services by domain affected - The Netherlands.

	**Exposed cohort**		**Unexposed cohort**	
	Support in standard school (%)	*Special school (%)*	*Support in standard school (%)*	*Special school (%)*
Cognition	0·0	10·8	0·7	10·1
Vision	0·0	0·0	0·0	0·0
Hearing	8·0	5·4	0·7	0·7
Behaviour	8·0	5·4	8·2	17·6
Speech difficulties	4·0	5·4	6·8	6·1
Physical	0·0	0·0	2·7	0·7
Other	4·0	2·7	0·0	0·0
Multi-domain	4·0	29·7	2·0	7·4
Reason not applicable	72·0	40·5	78·9	57·4
**Total**	100	100	100	100

Percentages in the Table refer to children in each cohort with a specific level of educational need (support in standard school or special school).

Table S5. Potential study biases in the assessments of exposure and outcome.

	Denmark		The Netherlands	
**Potential sources of biases**	**Bias**	**Direction**	**Bias**	**Direction**
Identification of exposed cohort	ICD codes have imperfect PPV for iGBS, as clinical diagnosis might have been presumed rather than microbiologically confirmed.	Outcome data might correspond to outcomes of a mix of truly GBS-exposed children and children exposed to other neonatal infections. Difficult to predict bias direction.<br><br>If misdiagnosis of iGBS is associated with mortality, this will bias the association between mortality and iGBS.	A non-negligible fraction of iGBS patients has culture-negative results[18-20] and might have been missed by our data capture approach.	The study was not designed to capture all invasive GBS exposed patients in NL. There would be a bias in estimates of outcomes if culture positivity at time of diagnosis were linked to future risk of NDI or mortality.
			Late-onset patients were less likely to be included in one of the datasets used to identify children with history of iGBS, because the perinatal registry is based on perinatal care supplied until 6 weeks of age. Therefore patients with disease onset between 6 weeks and 3 months are likely not to have been captured.	Children with history of early-onset iGBS might be over-represented in the exposed cohort. However, 36.1% of children with iGBS were late-onset, similar to findings in previous studies.
Determination of gestational age (GA)	Data on GA were missing for 28 neonates. These children were not included in the main analysis. However, they were included in the sensitivity analysis.	If children with early mortality were more likely to have missing GA data, due to difficulty in linkage between datasets, early mortality might have been underestimated.		

Age of onset of iGBS	The age of onset of iGBS was defined based on admission date. For infants who developed iGBS during hospitalization due to another cause (e.g. prematurity), the age of iGBS onset used in the analysis would not correspond to the actual age of symptoms onset.	Infants hospitalized in the first week of life due to another clinical condition and who developed iGBS during the same hospitalization might have been misclassified as early-onset iGBS. This could have impacted the early/late-onset stratified analyses.		
Neurodevelopmental impairment definition	NDI data in Denmark were restricted to patients diagnosed with NDI in a hospital/outpatient clinic setting.<br><br>Difficulty in categorizing NDI severity for a subset of ICD codes. These are presented as 'Non-categorized' in **Figure 2**.	This might lead to an underestimation of vision and hearing problems.<br><br>The proportions of children with iGBS with moderate or severe NDI might have been underestimated.	Special education needs were used as surrogate measure of NDI.	Mild NDI or severe NDI that does not result in enrollment in special needs schools might have been missed. Risk of NDI post-GBS might have been underestimated.

PPV, positive predictive value; iGBS, invasive GBS disease; NDI, neurodevelopmental impairment; GA, gestational age; DK, Denmark; NL, the Netherlands.

Supplementary Figures

Figure S1. Kaplan-Meier survival curves for children with invasive GBS disease compared to gestational age-matched unexposed children

A. Denmark

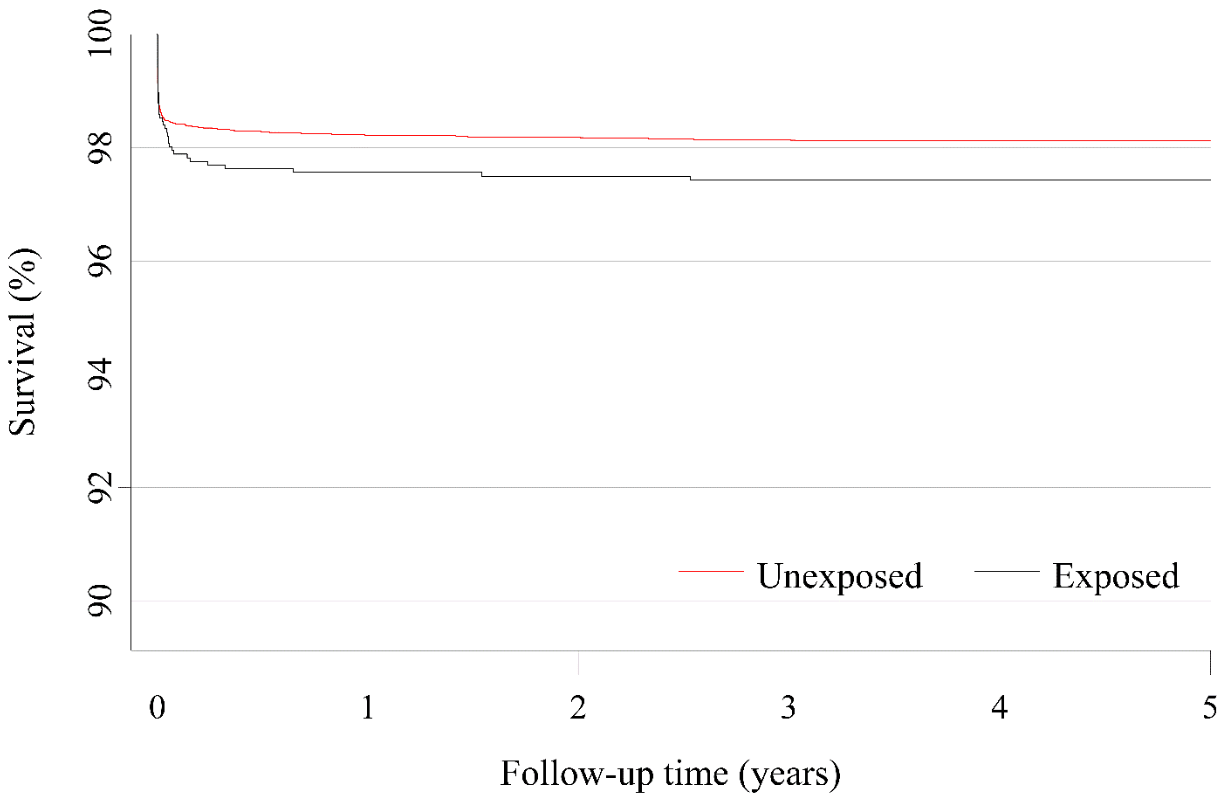

Number at risk:

GBS +	1561	1517	1461	1401	1344
GBS -	15501	15195	14669	14069	13524

B. The Netherlands

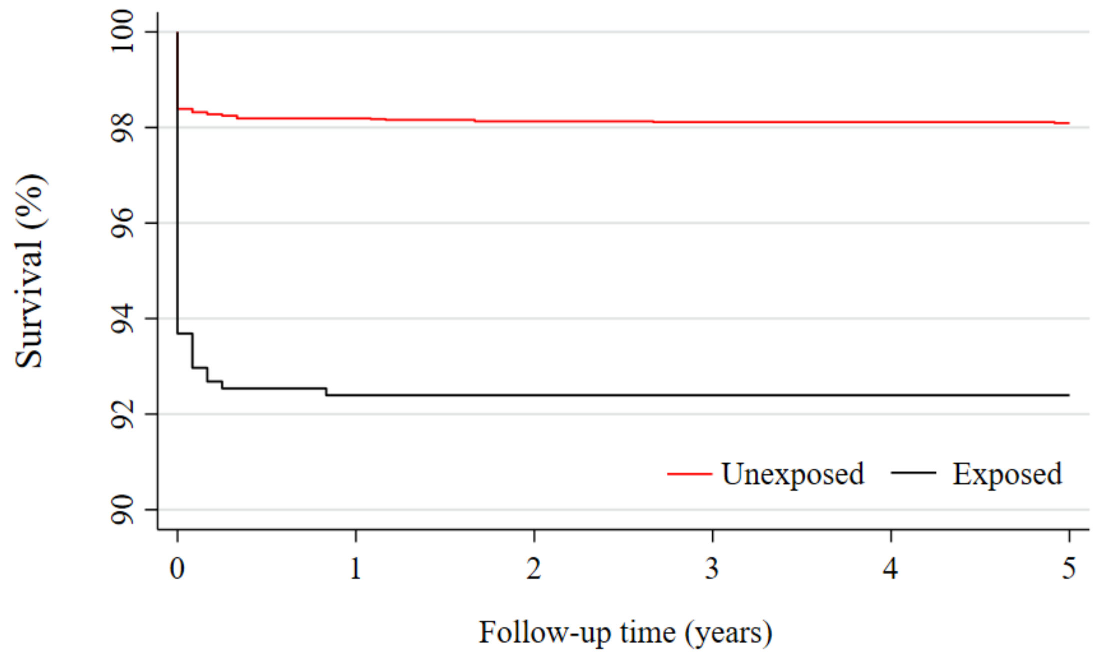

Number at risk:

GBS +	697	643	589	544	496
GBS -	6961	6834	6242	5747	5263

Figure S2. Household income in families of exposed (purple bars) and unexposed (orange bars) children.

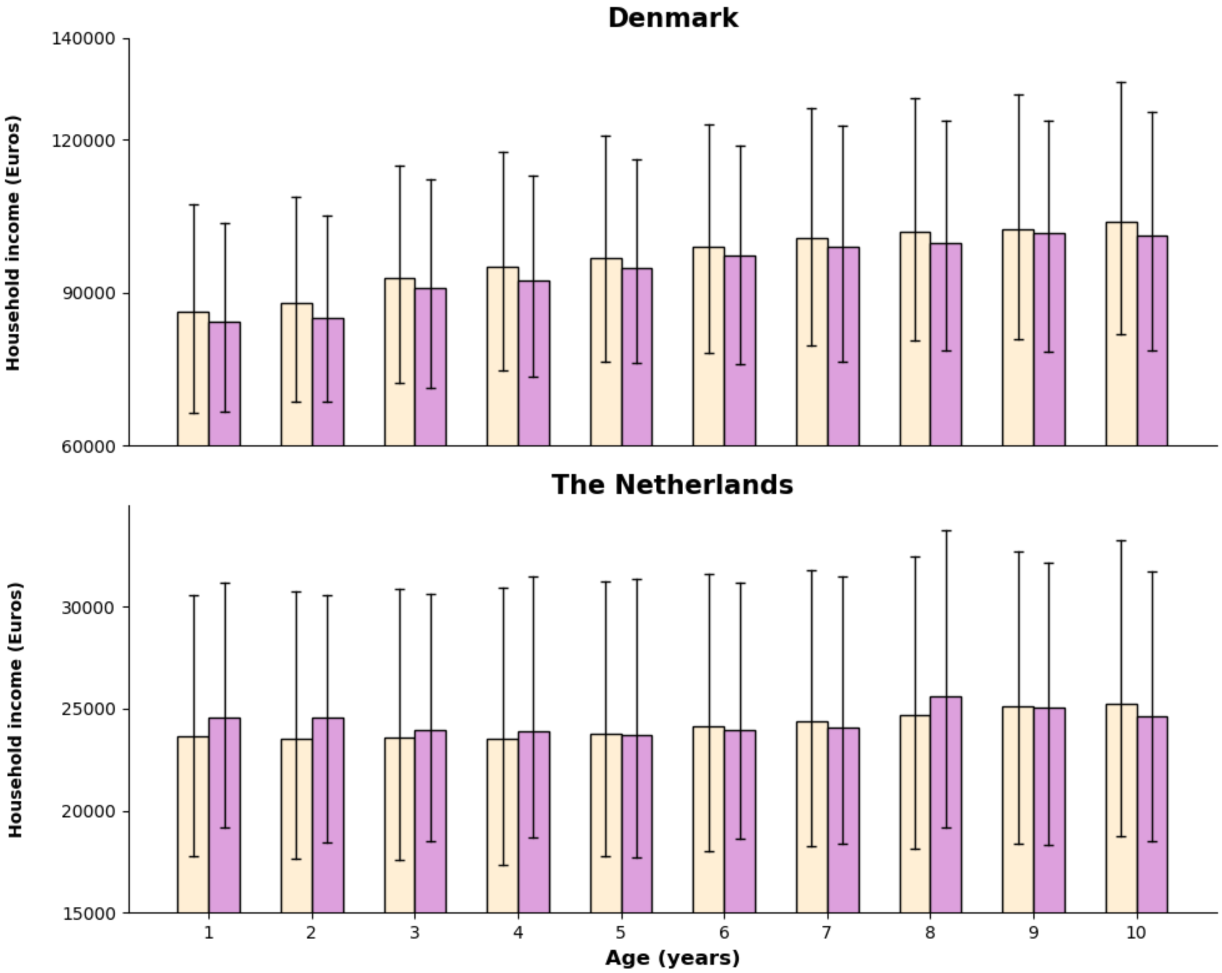

In both panels, y-axes represent household income, as described in the *Methods* section, with interquartile ranges, and x-axes show the age of exposed and unexposed children (or equivalently, time since the invasive GBS disease episode). In the Netherlands, data from exposed children who died in the first four months of life, data from matched unexposed children, and data from unexposed children who died during the same period were not included in the figure. In Denmark, the Income Statistics Register, which includes variables related to individual income, was used to derive annual household income, defined as the sum of the gross income of a cohort member's parents. Gross income for each year was measured before deductions for labor market and special pension contributions. In the Netherlands, household income was ascertained from a registry database of standardized disposable household income, after adjustment for family size and taxes. In both countries, incomes were adjusted to the year 2018 using the country-specific World Bank gross domestic product deflator. Danish income values then were converted from Krone to Euros.

Figure S3. Welfare received by households of exposed (purple bars) and unexposed (orange bars) children in the Netherlands.

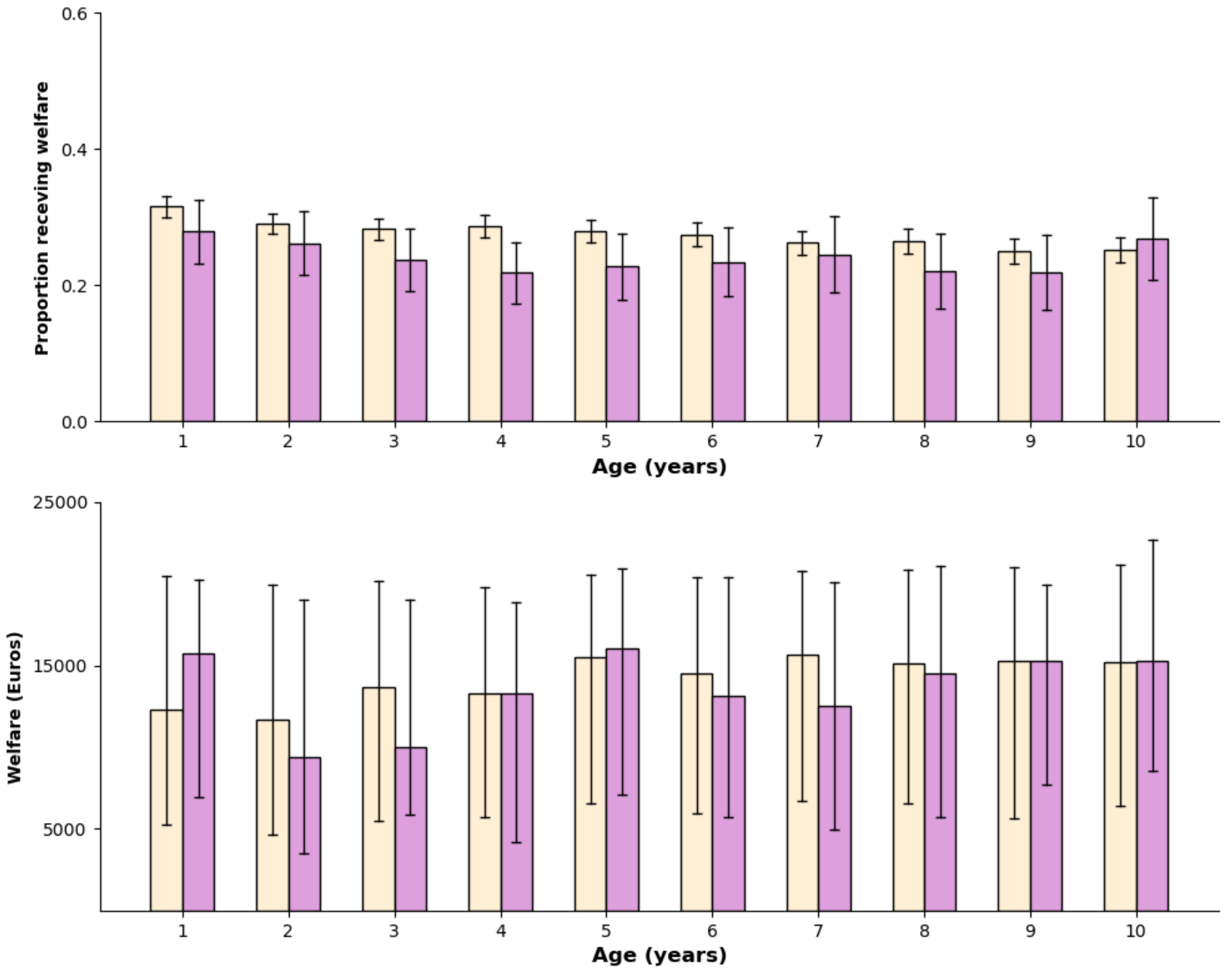

In the upper panel, the proportions, and confidence intervals, of families receiving welfare are shown. In the bottom panel, only data from families of children who received welfare are included (i.e., zeroes are excluded) and median welfare received and interquartile ranges are presented.

References

1. Schmidt M, Schmidt SAJ, Adelborg K, Sundboll J, Laugesen K, Ehrenstein V, et al. The Danish health care system and epidemiological research: from health care contacts to database records. *Clin Epidemiol* 2019; **11**: 563-91.
2. Schmidt M, Pedersen L, Sorensen HT. The Danish Civil Registration System as a tool in epidemiology. *Eur J Epidemiol* 2014; **29**(8): 541-9.
3. Bliddal M, Broe A, Pottegard A, Olsen J, Langhoff-Roos J. The Danish Medical Birth Register. *Eur J Epidemiol* 2018; **33**(1): 27-36.
4. Cohen E, Horvath-Puho E, Ray JG, Pedersen L, Adler N, Ording AG, et al. Association Between the Birth of an Infant With Major Congenital Anomalies and Subsequent Risk of Mortality in Their Mothers. *JAMA* 2016; **316**(23): 2515-24.
5. Sorensen HT, Horvath-Puho E, Pedersen L, Baron JA, Prandoni P. Venous thromboembolism and subsequent hospitalisation due to acute arterial cardiovascular events: a 20-year cohort study. *Lancet* 2007; **370**(9601): 1773-9.
6. Sundboll J, Horvath-Puho E, Adelborg K, Schmidt M, Pedersen L, Botker HE, et al. Higher Risk of Vascular Dementia in Myocardial Infarction Survivors. *Circulation* 2018; **137**(6): 567-77.
7. Schmidt M, Schmidt SA, Sandegaard JL, Ehrenstein V, Pedersen L, Sorensen HT. The Danish National Patient Registry: a review of content, data quality, and research potential. *Clin Epidemiol* 2015; **7**: 449-90.
8. Mors O, Perto GP, Mortensen PB. The Danish Psychiatric Central Research Register. *Scand J Public Health* 2011; **39**(7 Suppl): 54-7.
9. Baadsgaard M, Quitzau J. Danish registers on personal income and transfer payments. *Scand J Public Health* 2011; **39**(7 Suppl): 103-5.
10. World Bank national accounts data, and OECD National Accounts data files. Economic Policy & Debt: National accounts: Shares of GDP & other; 2020.
11. European Central Bank Statistical Data Warehouse. 2020.
12. Faber MJ, Burgers JS, Westert GP. A sustainable primary care system: lessons from the Netherlands. *J Ambul Care Manage* 2012; **35**(3): 174-81.
13. Netherlands Reference Laboratory For Bacterial Meningitis (AMC/RIVM). Bacterial meningitis in The Netherlands: Annual Report 2016. Amsterdam: University of Amsterdam; 2017.
14. Bijlsma MW, Bekker V, Brouwer MC, Spanjaard L, van de Beek D, van der Ende A. Epidemiology of invasive meningococcal disease in the Netherlands, 1960-2012: an analysis of national surveillance data. *Lancet Infect Dis* 2014; **14**(9): 805-12.
15. Statistics Netherlands. StatLine, The Hague/Heerlen, 2017 (Assessed October 24, 2017, at http://www.cbs.nl)
16. Eskes M, Ensing S, Groenendaal F, Abu-Hanna A, Ravelli A. The risk of intrapartum/neonatal mortality and morbidity following birth at 37 weeks of gestation: a nationwide cohort study. *BJOG* 2019; **126**(10): 1252-57.
17. PeriNed. Perinatale Zorg in Nederland (2000-2017) [Annual reports (2000-2017) PeriNed]. Utrecht; 2019.
18. de Gier B, van Kassel MN, Sanders EAM, van de Beek D, Hahné SJM, van der Ende A, et al. Disease burden of neonatal invasive Group B Streptococcus infection in the Netherlands. *PloS one* 2019; **14**(5): e0216749.
19. Trijbels-Smeulders M, de Jonge GA, Pasker-de Jong PC, Gerards LJ, Adriaanse AH, van Lingen RA, et al. Epidemiology of neonatal group B streptococcal disease in the Netherlands before and after introduction of guidelines for prevention. *Arch Dis Child Fetal Neonatal Ed* 2007; **92**(4): F271-6.
20. Trijbels-Smeulders M, Gerards LJ, M PC, de Jong P, van Lingen RA, Adriaanse AH, et al. Epidemiology of neonatal group B streptococcal disease in The Netherlands 1997-98. *Paediatr Perinat Epidemiol* 2002; **16**(4): 334-41.
